# Spirulina (*Arthrospira platensis*) Influences Nutritional Characteristics and the Relative GC–MS Chemical Profile of Aquaponically Cultivated Celery (*Apium graveolens*)

**DOI:** 10.3390/plants15192985

**Published:** 2026-09-30

**Authors:** Jehad Kalaji, Nermen Fekri, Mohamed M. Mahmoud, Mostafa B. Abouelela, Rania I. Nadeem, Khaled El-Adl, Sameh AbouZid, Asmaa I. Owis

**Affiliations:** 1 Pharmacognosy Department, Faculty of Pharmacy, Heliopolis University, Cairo 11785, Egypt; 2 Pharmacognosy Department, Faculty of Pharmacy, Beni-Suef University, Beni-Suef 62514, Egypt; 3 Agricultural Engineering Research Institute (AEnRI), Agricultural Research Center, Giza 12619, Egypt; 4 Pharmacognosy Department, Faculty of Pharmacy, Egyptian Russian University, Badr City 11829, Egypt; 5 Pharmacology and Toxicology Department, Faculty of Pharmacy, Heliopolis University, Cairo 11785, Egypt; 6 Pharmaceutical Medicinal Chemistry and Drug Design Department, Faculty of Pharmacy (Boys), Al-Azhar University, Nasr City 11884, Egypt

**Keywords:** aquaponics, *Arthrospira platensis*, *Apium graveolens*, biostimulant, GC-MS metabolite profiling, specialized metabolites, sustainable agriculture, phytochemical quality

## Abstract

Aquaponic cultivation provides a resource-efficient approach to crop production, while plant biostimulants may improve crop performance and nutritional quality. This study evaluated the effects of *Arthrospira platensis* (Spirulina) supplementation on celery (*Apium graveolens*) cultivated under aquaponic conditions. Aquaponic celery supplemented with 2, 4, or 8 g L^−1^ of Spirulina (CAS2, CAS4, and CAS8, respectively) was evaluated over three weeks. Untreated aquaponic celery (CA) was included as a week-3 endpoint reference, while soil-grown celery (CS) was included as a descriptive week-3 reference. Growth, biomass, protein, total lipids, crude fiber, mineral composition (Na, K, Ca, P, and Fe), and aflatoxin occurrence were assessed, and specialized metabolite profiles were characterized descriptively by gas chromatography–mass spectrometry (GC–MS). Among the supplemented groups, Spirulina dose was associated with changes in growth, biomass, nutritional composition, and mineral accumulation, with the magnitude of several responses varying across the three-week cultivation period. Because CA was sampled only at week 3, the longitudinal analyses evaluated dose-associated responses among CAS2, CAS4, and CAS8 and did not constitute a longitudinal comparison between Spirulina supplementation and the untreated control. At week 3, several growth and nutritional responses were higher in Spirulina-supplemented celery than in CA, with the greatest values for several responses generally observed at 8 g L^−1^. Potassium showed a non-monotonic response, with the highest week-3 value observed at 4 g L^−1^, whereas sodium increased markedly at higher supplementation levels. Descriptive GC–MS profiling showed differences in the relative representation of detected chemical classes among the analyzed samples; however, these profiles were obtained from representative samples without independent biological replication and were therefore used for descriptive chemical characterization rather than inferential testing. No aflatoxins were detected. Overall, Spirulina supplementation was associated with dose- and time-dependent differences in growth and selected nutritional and mineral characteristics among the supplemented aquaponic celery groups, while week-3 comparisons indicated higher values for several responses relative to untreated aquaponic celery. The GC–MS analysis provided descriptive evidence of differences in the relative chemical profiles of the analyzed samples.

## 1. Introduction

Global agriculture is increasingly challenged by climate change, rapid population growth, freshwater scarcity, and environmental degradation, all of which threaten the sustainability of food production systems. Recent reports indicate that billions of people experience water stress or severe water scarcity each year, and increasing pressure on freshwater resources is expected to intensify the need for water-efficient agricultural technologies [1]. Consequently, developing sustainable crop production systems capable of simultaneously improving resource-use efficiency, crop quality, and environmental sustainability has become a global priority. Achieving the United Nations Sustainable Development Goal 6 (SDG 6), which aims to ensure the availability and sustainable management of water and sanitation for all, further emphasizes the importance of adopting innovative cultivation strategies for sustainable agriculture [2].

Aquaponics has emerged as one of the most promising sustainable crop production systems because it integrates aquaculture and hydroponics into a recirculating, closed-loop ecosystem. In this integrated system, nutrient-rich fish wastes are biologically mineralized by nitrifying microorganisms into plant-available nutrients, substantially reducing freshwater consumption, dependence on synthetic fertilizers, and nutrient discharge into the environment, thereby improving resource-use efficiency. Consequently, aquaponics represents an environmentally sustainable cultivation strategy, particularly suitable for arid and semi-arid regions, including Egypt, where freshwater resources are increasingly limited. Previous studies have demonstrated that aquaponically cultivated crops can achieve growth performance and product quality comparable to conventionally cultivated plants while using considerably less water and supporting circular bioeconomy principles [3,4,5].

Despite these advantages, optimizing nutrient availability, crop productivity, and product quality remains a major challenge in aquaponic systems. In this context, plant biostimulants have attracted considerable attention as environmentally friendly agricultural inputs capable of improving nutrient use efficiency, abiotic stress tolerance, crop quality, and nutrient availability. Du Jardin first established the modern scientific framework for plant biostimulants, defining them as substances or microorganisms that improve nutrient use efficiency, abiotic stress tolerance, and crop quality regardless of their nutrient content [6]. This concept was subsequently incorporated into Regulation (EU) 2019/1009, which defines plant biostimulants as products that stimulate plant nutritional processes independently of their nutrient content with the objective of improving nutrient use efficiency, tolerance to abiotic stress, quality traits, or nutrient availability in the rhizosphere [7,8].

Among naturally derived biostimulants, the filamentous cyanobacterium *Arthrospira platensis* (commercially known as Spirulina) has emerged as a promising candidate because of its exceptional nutritional and biochemical composition. Spirulina contains approximately 30–70% protein (dry weight) together with essential amino acids, vitamins, minerals, pigments, polysaccharides, fatty acids, and numerous bioactive metabolites. These constituents collectively contribute to its biostimulant activity by enhancing nutrient acquisition and assimilation, improving photosynthetic performance, strengthening defense systems, and stimulating specialized metabolism. Previous studies have demonstrated that Spirulina-based formulations enhance plant growth, nutrient uptake, and tolerance to abiotic stress, highlighting their potential as sustainable biostimulants for environmentally friendly agricultural production. Beyond improving plant growth, recent evidence suggests that microalgal biostimulants can reprogram specialized metabolism by modulating specialized metabolic pathways, particularly terpenoid, phenylpropanoid, and lipid metabolism, thereby enhancing the accumulation of bioactive metabolites and improving crop nutritional quality [9,10,11,12].

Among economically important medicinal and vegetable crops, celery (*Apium graveolens* L.) is widely recognized for its high nutritional value and diverse phytochemical composition. Celery is particularly valued because it is one of the richest dietary sources of phthalides, together with terpenoids, phenolic acids, flavonoids, coumarins, and other specialized metabolites that collectively determine its characteristic aroma, nutritional quality, and diverse biological activities, including antioxidant, anti-inflammatory, antimicrobial, antihypertensive, and cardioprotective effects. Recent advances in GC–MS- and LC–MS-based metabolomic analyses have revealed remarkable chemical diversity in celery and demonstrated that genotype, environmental conditions, and cultivation practices markedly influence its metabolite profile and biological activities. These findings have highlighted the importance of optimizing cultivation practices not only to maximize crop productivity but also to enhance phytochemical composition and nutritional quality. Among these metabolites, phthalides constitute the characteristic chemical markers of celery, whereas diterpenes, phytosterols, and tocopherols contribute substantially to its nutritional and pharmaceutical value, making them suitable targets for evaluating the effects of biostimulant application [3,13].

The nutritional quality of celery, including its protein, lipid, dietary fiber, and essential mineral contents (potassium, calcium, phosphorus, iron, and sodium), is strongly influenced by nutrient availability and cultivation practices. Because aquaponic systems continuously recycle dissolved nutrients, they provide an efficient platform for improving nutrient use efficiency while reducing dependence on external fertilizer inputs. Supplementation with Spirulina may further enhance plant physiological performance, nutrient assimilation, and specialized metabolism through the supply of amino acids, minerals, vitamins, pigments, polysaccharides, and other bioactive constituents. Previous studies have demonstrated that natural biostimulants improve nutrient acquisition, crop quality, and resilience to abiotic stress, supporting their application in sustainable crop production. However, relatively little is known about how Spirulina supplementation influences the coordination between primary metabolism (nutritional constituents) and specialized metabolism [8,14,15,16].

In addition to nutritional quality, food safety is a fundamental component of sustainable agricultural production. Aflatoxins produced by *Aspergillus* species are among the most hazardous mycotoxins contaminating agricultural commodities and pose serious risks to both human and animal health. Previous studies have reported antimicrobial and antioxidant activities of Spirulina; however, its potential contribution to reducing fungal contamination and mycotoxin occurrence in crop production systems remains insufficiently investigated and requires further evaluation [17,18].

Although Spirulina has been investigated as a natural biostimulant in several agricultural systems, its application in aquaponic cultivation remains poorly explored, particularly for medicinal and aromatic vegetables such as celery. Moreover, most previous studies have focused primarily on growth promotion, nutrient uptake, or antioxidant activity, whereas comprehensive investigations integrating GC–MS-based metabolite profiling with nutritional evaluation, mineral profiling, and food safety assessment within a single experimental framework remain scarce. Consequently, the mechanisms by which Spirulina remodels specialized metabolism and influences the accumulation of characteristic celery metabolites, including phthalides, diterpenes, phytosterols, and tocopherols, remain poorly understood [3].

Metabolomic profiling has become a powerful approach for elucidating plant responses to cultivation practices and biostimulant application because it provides a comprehensive overview of coordinated changes in specialized metabolism rather than isolated alterations in individual metabolites. Such analyses can reveal metabolic pathways associated with improved crop quality and nutritional value that may not be evident through conventional phytochemical analyses alone. In particular, heatmap visualization and metabolite-class analysis facilitate the interpretation of coordinated biochemical responses associated with cultivation practices and biostimulant application [3].

Therefore, this study aimed to evaluate the effects of Spirulina supplementation at 2, 4, and 8 g L^−1^ on the growth, nutritional composition (protein, crude fiber, and total lipids), mineral accumulation (Na, K, Ca, P, and Fe), and aflatoxin occurrence of aquaponically cultivated celery, and to characterize the associated relative GC–MS metabolite profiles. Longitudinal responses were evaluated among the three Spirulina-supplemented groups (CAS2, CAS4, and CAS8), whereas comparisons with untreated aquaponic celery (CA) were restricted to week-3 endpoint measurements. A secondary objective was to descriptively compare the relative GC–MS metabolite-class profiles of Spirulina-supplemented and untreated aquaponic celery with those of soil-grown celery at week 3, recognizing that the soil-grown plants were cultivated under different environmental and management conditions.

We hypothesized that repeated supplementation with increasing nominal concentrations of *A. platensis* (2, 4, and 8 g L^−1^) would be associated with dose- and time-related differences in vegetative growth, biomass production, nutritional composition, and mineral accumulation among the Spirulina-supplemented aquaponic celery groups. We further hypothesized that week-3 endpoint measurements would differ between Spirulina-supplemented and untreated aquaponic celery and that Spirulina supplementation would be associated with differences in the relative GC–MS metabolite profile. Because soil-grown celery was sampled only at week 3 and was cultivated under different environmental and management conditions, comparisons with the soil-grown control were considered contextual and descriptive rather than a direct test of the primary treatment hypothesis.

## 2. Results and Discussion

### 2.1. Model Diagnostics

Model assumptions were evaluated for all inferential analyses. The growth mixed-effects models for leaf length and leaf width converged successfully and were not classified as singular. The estimated plant-level random-intercept variances were 0.02333 for leaf length and 0.02500 for leaf width. Residual-versus-fitted and normal Q–Q diagnostics did not indicate substantial departures from the assumed model structure. For the destructive measurements, preliminary mixed-effects models including experimental repetition as a random effect produced boundary or near-zero random-effect variance estimates for several response variables. Because experimental repetition had only three levels, repetition was therefore modeled as a fixed blocking factor in the final destructive analyses. This approach avoids unstable estimation of a random-effect variance from only three levels while still accounting for systematic differences among experimental repetitions. Because CA and CS were sampled only at week 3, they were excluded from the longitudinal W1–W3 destructive analyses; these analyses therefore included only CAS2, CAS4, and CAS8. Most nutritional and mineral responses showed acceptable residual diagnostics. Evidence of nonconstant residual variance was identified for fresh biomass and dry biomass in the longitudinal analyses and for week-3 iron. These responses were therefore subjected to HC3 heteroscedasticity-robust sensitivity analysis and interpreted cautiously. Dry matter showed essentially zero residual variance in the longitudinal model and was therefore not used for formal inferential interpretation. Accordingly, dry matter was not included in the HC3 sensitivity analyses. Influence diagnostics were examined using studentized residuals and Cook’s distance. Although some observations showed moderate influence, no observation was excluded solely on the basis of statistical influence because there was no independent experimental or analytical justification for its removal. Because only three independent biological replicates were available per treatment, formal normality and variance tests were interpreted together with graphical diagnostics rather than used as the sole basis for accepting or rejecting model assumptions.

### 2.2. Growth and Biomass Accumulation

#### 2.2.1. Growth Evaluation

**Figure 1:**
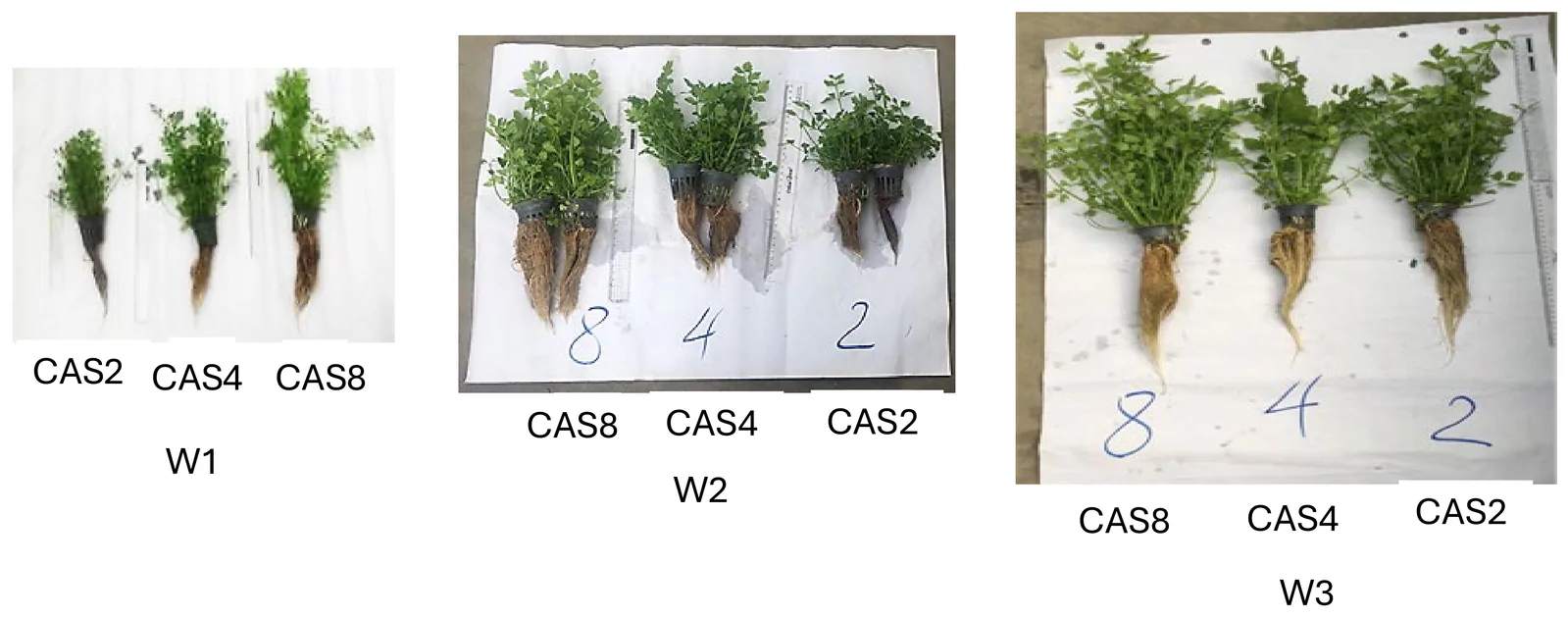
Growth of aquaponically cultivated celery during the first three weeks of Spirulina supplementation. Longitudinal comparisons represent Spirulina-dose effects among CAS2, CAS4, and CAS8; the untreated aquaponic control (CA) was sampled only at week 3.

**Figure 2:**
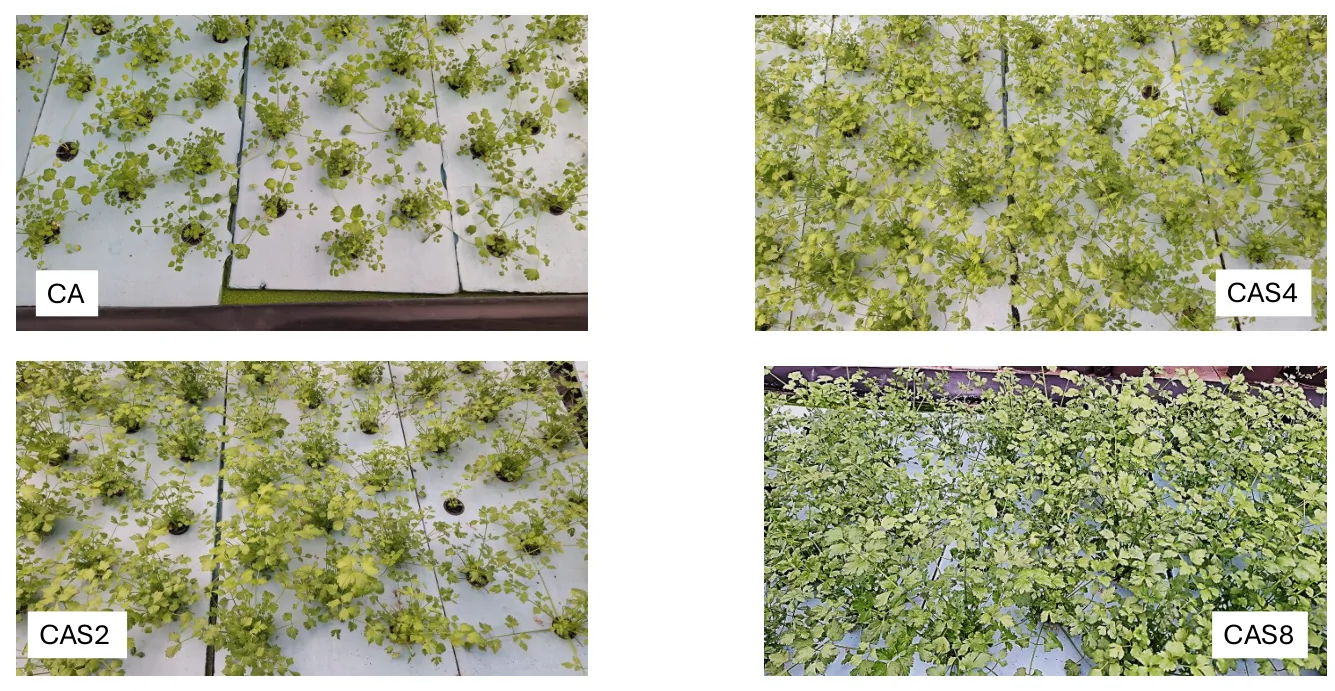
Representative aquaponic celery plants after three weeks of cultivation under the respective treatments. CA = untreated aquaponic control; 2 = 2 g L^−1^ Spirulina; 4 = 4 g L^−1^ Spirulina; 8 = 8 g L^−1^ Spirulina.

**Figure 3:**
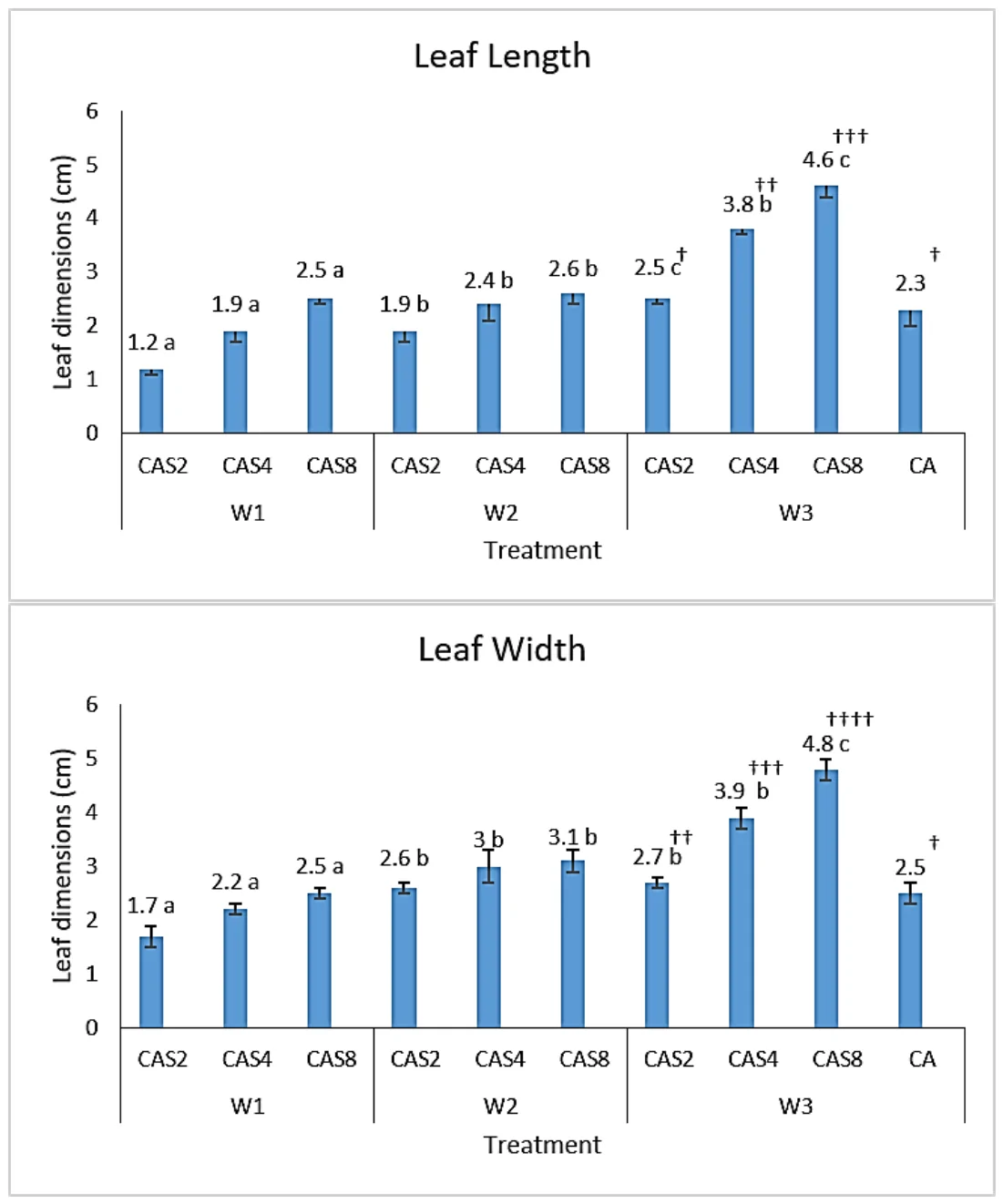
Leaf dimensions of aquaponically cultivated celery following Spirulina supplementation. Leaf length and leaf width were measured after one (W1), two (W2), and three (W3) weeks of supplementation with 2, 4, and 8 g L^−1^ of Spirulina. Values are presented as mean ± SD (n = 3 independent tank-level biological replicates). Different letters within each week indicate significant differences among Spirulina doses based on Tukey-adjusted simple-effect comparisons following a significant Spirulina dose × week interaction (*p* < 0.05). The untreated aquaponic control (CA) was sampled only at week 3 and was therefore not included in these longitudinal comparisons. ^†^ *p* < 0.05; ^††^ *p* < 0.01; ^†††^ *p* < 0.001; ^††††^ *p* < 0.0001, based on the prespecified Dunnett comparisons between the Spirulina-supplemented treatments (CAS2, CAS4, and CAS8) and the untreated aquaponic control (CA) at week 3.

The effect of *A. platensis* supplementation on the vegetative growth of aquaponically cultivated celery was evaluated by measuring leaf length and width weekly over three weeks (W1–W3) (Table 1; Figure 1, Figure 2 and Figure 3). Because the same individually identified plants were followed throughout the three-week period, measurements at W1–W3 represented repeated observations. The growth data were therefore analyzed using a linear mixed-effects model incorporating Spirulina dose, sampling week, and Spirulina dose × week interaction as fixed effects, with plant identity as the random intercept only.

Leaf dimensions increased progressively over the three-week cultivation period in the Spirulina-treated groups. At week 3, the soil-grown control (CS) showed leaf length and width of 2.0 and 2.1 cm, respectively, whereas untreated aquaponic celery (CA) showed values of 2.3 and 2.5 cm, respectively. Among the Spirulina-treated groups, CAS8 showed the highest numerical values at week 3, reaching 4.6 cm in leaf length and 4.8 cm in leaf width, followed by CAS4, with corresponding values of 3.8 and 3.9 cm. During the first week, leaf length ranged from 1.2 cm in CAS2 to 2.5 cm in CAS8, while leaf width ranged from 1.7 to 2.5 cm, respectively. At week 2, CAS8 reached 2.6 cm in leaf length and 3.1 cm in leaf width. At the week-3 endpoint, blocked one-way ANOVA showed significant differences among the four aquaponic groups for leaf length (F_3,6_ = 1074.00, *p* < 0.0001) and leaf width (F_3,6_ = 1395.00, *p* < 0.0001).

Leaf length and leaf width showed significant Spirulina dose × week interactions among the supplemented groups (CAS2, CAS4, and CAS8), indicating that the magnitude of the dose-associated differences varied across sampling weeks. Because CA was sampled only at week 3, the longitudinal analysis did not provide a comparison between Spirulina supplementation and the untreated aquaponic control over time. Therefore, the longitudinal dose and week effects were interpreted using simple-effect comparisons among the three Spirulina doses rather than as evidence of a longitudinal Spirulina-versus-control effect. Estimated marginal means and pairwise contrasts were evaluated using Tukey adjustment with the emmeans package, with Tukey adjustment applied to the relevant comparison families.

For leaf length, the three Spirulina doses differed significantly within W1 and W3, whereas at W2 the CAS4–CAS8 comparison was not significant (estimated difference = −0.17 cm, 95% CI −0.52 to 0.18 cm, Tukey-adjusted *p* = 0.459). At W3, leaf length increased progressively with Spirulina dose, with estimated differences of 1.30 cm for CAS4 versus CAS2 (95% CI 0.95–1.65 cm, *p* < 0.001), 2.10 cm for CAS8 versus CAS2 (95% CI 1.75–2.45 cm, *p* < 0.001), and 0.80 cm for CAS8 versus CAS4 (95% CI 0.45–1.15 cm, *p* < 0.001). Within CAS2 and CAS4, all pairwise week comparisons were significant (*p* < 0.001). Within CAS8, W1 did not differ significantly from W2 (estimated difference = −0.10 cm, 95% CI −0.27 to 0.07 cm, *p* = 0.305), whereas W1 versus W3 and W2 versus W3 were significant (*p* < 0.001).

A similar Spirulina dose × week interaction was observed for leaf width. Within W1, CAS2 differed significantly from CAS4 (estimated difference = −0.50 cm, 95% CI −0.86 to −0.14 cm, *p* = 0.006) and CAS8 (−0.80 cm, 95% CI −1.16 to −0.44 cm, *p* < 0.001), whereas CAS4 versus CAS8 was not significant (*p* = 0.108). The same pattern was observed at W2, with CAS2 differing from CAS4 (−0.43 cm, 95% CI −0.79 to −0.08 cm, *p* = 0.016) and CAS8 (−0.50 cm, 95% CI −0.86 to −0.14 cm, *p* = 0.006), whereas CAS4 versus CAS8 was not significant (*p* = 0.883). At W3, all three Spirulina doses differed significantly from one another (all *p* < 0.001). Within CAS4 and CAS8, all pairwise week comparisons were significant (*p* < 0.001). Within CAS2, W1 differed significantly from W2 and W3, whereas W2 versus W3 was not significant (estimated difference = −0.10 cm, 95% CI −0.24 to 0.04 cm, *p* = 0.174). At the week-3 endpoint, blocked one-way ANOVA showed significant differences among the four aquaponic groups for leaf length (F_3,6_ = 1074.00, *p* < 0.0001) and leaf width (F_3,6_ = 1395.00, *p* < 0.0001). Tukey-adjusted comparisons indicated that CA, CAS2, CAS4, and CAS8 differed significantly from one another for both leaf length and leaf width (*p* < 0.05), with CAS8 showing the highest values, followed by CAS4, CAS2, and CA. Dunnett comparisons with CA, which were prespecified as the Spirulina-supplemented group versus untreated-control comparisons, showed significant differences for CAS2, CAS4, and CAS8 for both leaf length and leaf width (*p* < 0.05).

At the final harvest, the larger leaf dimensions observed in the Spirulina-treated groups, especially at 8 g L^−1^, compared with untreated aquaponic celery were observed under the conditions of the present experiment. The response is consistent with previous reports describing the ability of microalgal biostimulants to influence vegetative development and plant physiological performance. Microalgal preparations may contain nutrients, amino acids, vitamins, polysaccharides, pigments, and other bioactive constituents capable of affecting nutrient acquisition and plant metabolic processes [8,14,15]. These properties may contribute to the observed growth response; however, the present study did not directly measure the physiological pathways responsible for it. Mechanisms involving chlorophyll biosynthesis, photosynthetic carbon fixation, nitrogen assimilation, or activation of specific stress-response pathways were not directly investigated and therefore remain plausible explanations rather than demonstrated mechanisms [19,20].

#### 2.2.2. Fresh Biomass, Dry Biomass, and Dry Matter Content

**Figure 4:**
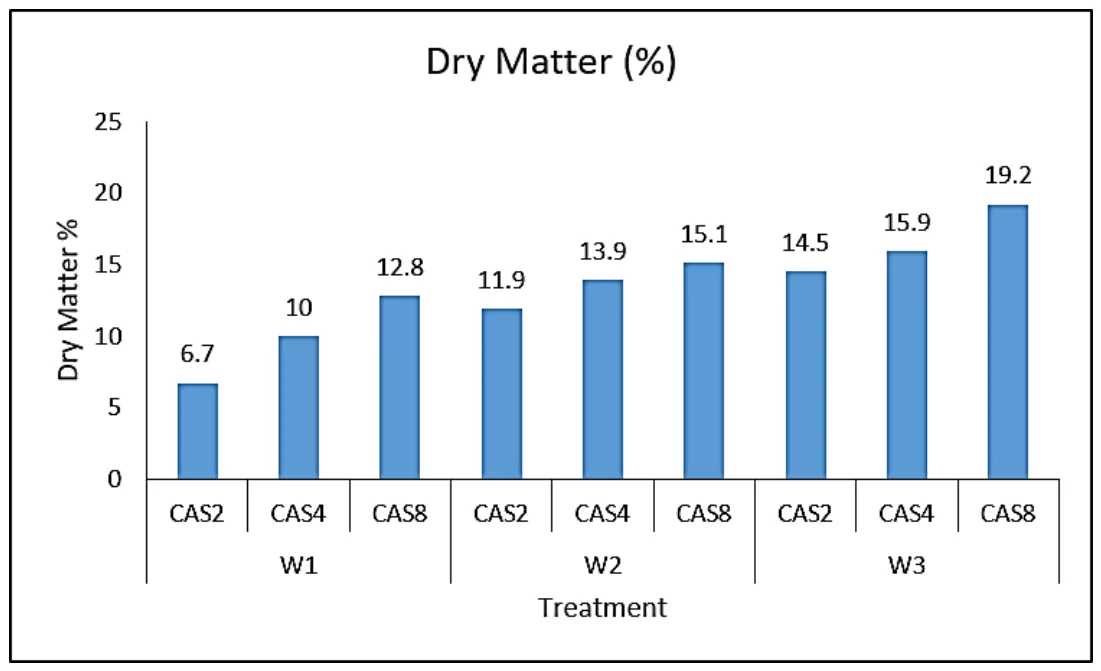
Dry matter percentage of aquaponically cultivated celery following Spirulina supplementation. Dry matter percentage was measured after one (W1), two (W2), and three (W3) weeks of supplementation with 2, 4, and 8 g L^−1^ of Spirulina. Different plants were harvested at each sampling week. Values are presented as mean ± SD (n = 3 independent tank-level biological replicates). Week-3 dry matter values are presented descriptively because the corresponding model showed essentially zero residual variance and did not provide stable inferential estimates. Longitudinal comparisons were restricted to CAS2, CAS4, and CAS8 because CA was sampled only at week 3.

The effects of Spirulina dose on fresh biomass, dry biomass, and dry matter content are presented in Table 2 and Figure 4. Because these measurements were obtained destructively from different plants at each sampling week, they were analyzed using the longitudinal linear-model approach described in the Section 3.15, with experimental repetition included as a fixed blocking factor. Because CA was sampled only at week 3, the longitudinal analysis only included CAS2, CAS4, and CAS8. Therefore, these analyses evaluated Spirulina-dose effects among the supplemented groups over time and did not test differences in temporal trajectories between supplemented and untreated aquaponic celery. At week 3, dry matter content was 19.2% in CAS8 and 17.7% in CA, while soil-grown celery showed 22.0%. The dry matter model showed essentially zero residual variance; therefore, formal inferential statistics for dry matter were not considered stable and the week-3 dry matter values are presented descriptively rather than as statistically significant group differences.

Fresh and dry biomass showed significant Spirulina dose × week interactions among the supplemented groups (CAS2, CAS4, and CAS8) in the longitudinal fixed-block linear models. Simple-effect comparisons showed that fresh biomass differed significantly among CAS2, CAS4, and CAS8 at every sampling week, and the magnitude of the dose differences increased over time. At W3, CAS4 exceeded CAS2 by 105.0 g (95% CI 97.5–112.5 g, Tukey-adjusted *p* < 0.001), CAS8 exceeded CAS2 by 149.8 g (95% CI 142.3–157.3 g, *p* < 0.001), and CAS8 exceeded CAS4 by 44.8 g (95% CI 37.3–52.3 g, *p* < 0.001). Within each dose, fresh biomass increased significantly across all sampling intervals (all *p* < 0.001). Dry biomass showed the same dose-associated and time-associated pattern among the supplemented groups. At W3, CAS4 exceeded CAS2 by 18.2 g (95% CI 17.2–19.2 g, *p* < 0.001), CAS8 exceeded CAS2 by 33.9 g (95% CI 32.9–34.9 g, *p* < 0.001), and CAS8 exceeded CAS4 by 15.7 g (95% CI 14.7–16.7 g, *p* < 0.001). All pairwise week comparisons were significant within each dose (*p* < 0.001). At the week-3 endpoint, blocked ANOVA showed significant differences among the four aquaponic groups for fresh biomass (F_3,6_ = 1642.08, *p* < 0.001) and dry biomass (F_3,6_ = 2759.66, *p* < 0.001). For fresh biomass, CA and CAS2 did not differ significantly, whereas CAS4 and CAS8 were higher than CA and CAS2, and CAS8 was higher than CAS4. For dry biomass, all four aquaponic groups differed significantly. Dunnett-adjusted comparisons showed significant differences between CA and CAS4 and CAS8 for fresh biomass and between CA and CAS2, CAS4, and CAS8 for dry biomass. Dry matter values are presented descriptively because the longitudinal model showed essentially zero residual variance and did not provide stable inferential estimates.

Sensitivity analysis using the HC3 heteroscedasticity-robust procedure gave the same qualitative conclusions for fresh and dry biomass. For fresh biomass, the treatment, week, and treatment × week effects remained significant (HC3 F = 51.78, 417.25, and 197.20, respectively; all *p* < 0.001), compared with the corresponding conventional F-values of 1642.08, 4471.38, and 239.64. For dry biomass, the treatment, week, and treatment × week effects also remained significant under HC3 (F = 54.45, 605.09, and 354.03, respectively; all *p* < 0.001), compared with conventional F-values of 2759.66, 6431.51, and 493.42. Thus, the HC3 sensitivity analysis did not change the interpretation of the longitudinal fresh- or dry-biomass results.

These findings indicate that biomass accumulation was associated with both Spirulina concentration and cultivation time among the supplemented groups. The higher doses were associated with progressively greater fresh and dry biomass among CAS2, CAS4, and CAS8, while the magnitude of the dose differences became larger toward W3. The increased biomass may be consistent with the nutrient and bioactive composition of Spirulina, which contains proteins, amino acids, minerals, vitamins, pigments, polysaccharides, and other compounds that may influence nutrient availability and plant metabolic processes. However, the present study did not directly measure nutrient uptake, photosynthetic activity, or the biochemical pathways responsible for biomass accumulation; therefore, these mechanisms remain hypotheses rather than demonstrated causal pathways [8,11,16].

Although soil-grown celery exhibited the highest dry matter percentage (22.0%), CAS8 produced substantially greater fresh and dry biomass, reaching 260.4 and 49.9 g plant^−1^, respectively, compared with 173.0 and 38.0 g plant^−1^ in soil-grown plants. The higher biomass observed in CAS8 therefore reflects greater overall biomass accumulation under the experimental conditions, rather than an increase in dry matter percentage. The dry matter values among the aquaponic treatments should be interpreted descriptively because the corresponding statistical model showed essentially zero residual variance and did not provide stable inferential estimates. The slightly lower dry matter percentage in the Spirulina-treated groups than in soil-grown celery may reflect differences in tissue water content or developmental status; however, water-content dynamics were not directly measured.

Consequently, despite a slightly lower dry matter percentage than soil-grown celery, CAS8 showed substantially greater fresh and dry biomass. This difference indicates higher biomass accumulation under the evaluated specific aquaponic conditions; however, it should not be attributed solely to the aquaponic cultivation system because the soil-grown plants were cultivated under different environmental and management conditions. The lower dry matter percentage of Spirulina-treated plants should therefore not be interpreted as evidence of lower biomass quality or reduced structural accumulation. Comparable improvements in fresh and dry biomass following Spirulina supplementation have been reported for horticultural crops, where microalgal preparations have been associated with improved plant growth and physiological performance [8,14,15,21].

Overall, these findings indicate that higher Spirulina doses were associated with greater biomass production among the supplemented groups, while the week-3 endpoint analysis additionally showed significant differences between selected Spirulina-supplemented groups and the untreated aquaponic control. The present experiment did not directly determine the physiological mechanisms responsible for this response or quantify changes in water use, energy consumption, fertilizer requirements, or production costs. Accordingly, the findings support a potential agronomic benefit of Spirulina supplementation under the tested conditions but do not by themselves establish improved sustainability or resource-use efficiency.

### 2.3. Nutritional Composition and Mineral Profiling

Because the untreated aquaponic control (CA) was sampled only at week 3, it was not included in the W1–W3 longitudinal models. Accordingly, all longitudinal Spirulina-dose and Spirulina-dose × week effects reported below represent differences among the three Spirulina-supplemented groups (CAS2, CAS4, and CAS8), rather than comparisons between supplemented and untreated aquaponic celery. The week-3 endpoint analyses, in contrast, included CA together with CAS2, CAS4, and CAS8 and therefore provide the direct comparison with the untreated aquaponic control at week 3.

#### 2.3.1. Protein, Crude Fiber, and Total Lipid Content

**Figure 5:**
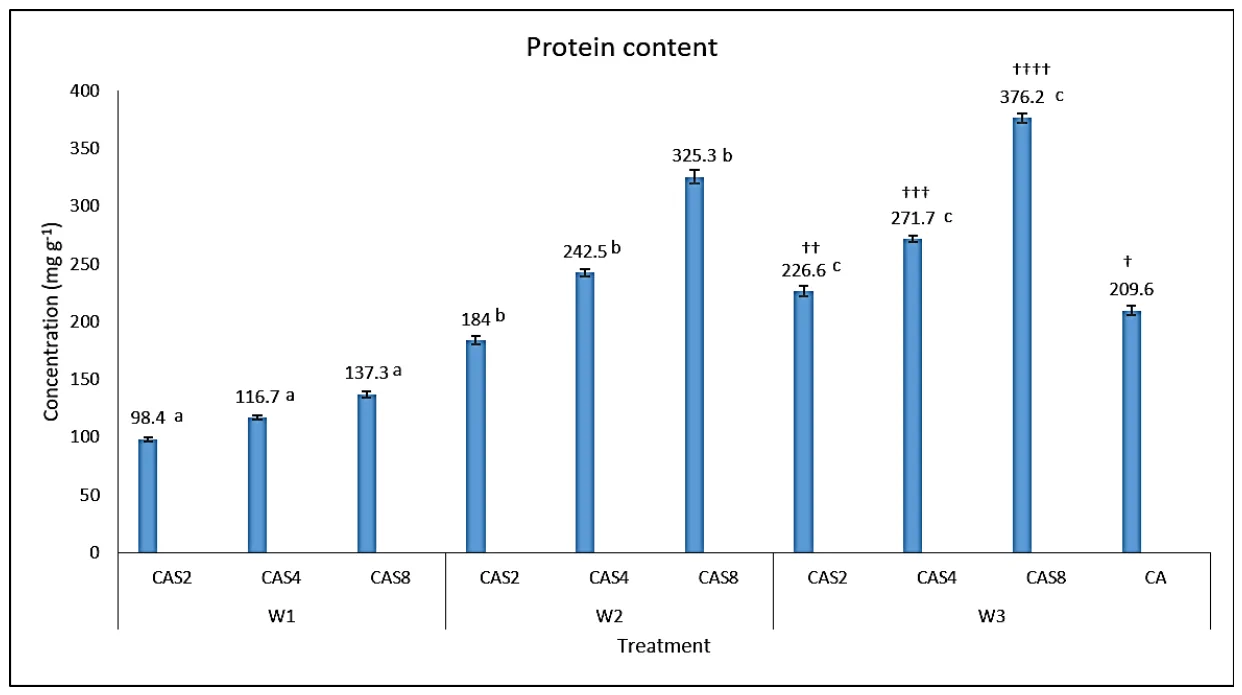
Protein content of aquaponically cultivated celery following Spirulina supplementation. Protein content was measured after one (W1), two (W2), and three (W3) weeks of supplementation with 2, 4, and 8 g L^−1^ of Spirulina. Values are presented as mean ± SD (n = 3 independent tank-level biological replicates). Different letters within each week indicate significant differences among Spirulina doses based on Tukey-adjusted simple-effect comparisons following a significant Spirulina dose × week interaction (*p* < 0.05). The longitudinal analysis only included CAS2, CAS4, and CAS8 because CA was sampled only at week 3. ^†^ *p* < 0.05; ^††^ *p* < 0.01; ^†††^ *p* < 0.001; ^††††^ *p* < 0.0001, based on the prespecified Dunnett comparisons between the Spirulina-supplemented treatments (CAS2, CAS4, and CAS8) and the untreated aquaponic control (CA) at week 3.

The effects of Spirulina dose on total protein content are presented in Table 3 and Figure 5. Total protein content increased across Spirulina concentrations and sampling weeks, with the highest observed value at week 3. Among the aquaponic groups, the highest protein content was recorded in celery supplemented with 8 g L^−1^ Spirulina (CAS8) after three weeks (376.2 ± 4.0 µg g^−1^ FW), followed by CAS4 (271.7 ± 3.0 µg g^−1^ FW) and CAS2 (226.6 ± 4.3 µg g^−1^ FW). Untreated aquaponic celery also contained substantially more protein (209.6 ± 4.3 µg g^−1^ FW) than the soil-grown plants (92.2 ± 2.7 µg g^−1^ FW). This difference suggests that the aquaponic plants accumulated more protein under the conditions evaluated; however, the difference cannot be attributed solely to aquaponic cultivation because the soil-grown plants were cultivated under different environmental and management conditions and were sampled only at week 3. Compared with untreated aquaponic celery, Spirulina supplementation increased protein content by approximately 8.1%, 29.6%, and 79.5% in CAS2, CAS4, and CAS8, respectively, after three weeks, whereas CAS8 showed the highest protein content among the aquaponic groups at week 3 (376.2 ± 4.0 µg g^−1^ FW). The soil-grown value is presented separately as a contextual reference. The longitudinal analysis showed significant Spirulina-dose, week, and Spirulina-dose × week effects. Because the interaction was significant, simple-effect comparisons were used to characterize the dose-associated differences within each week and temporal changes within each dose. Protein differed significantly among CAS2, CAS4, and CAS8 at every sampling week, and the magnitude of the differences increased over time. At W3, CAS4 exceeded CAS2 by 45.19 µg g^−1^ FW (95% CI 40.03–50.34, Tukey-adjusted *p* < 0.001), CAS8 exceeded CAS2 by 149.67 µg g^−1^ FW (95% CI 144.52–154.83, *p* < 0.001), and CAS8 exceeded CAS4 by 104.49 µg g^−1^ FW (95% CI 99.33–109.64, *p* < 0.001). Protein increased significantly between each pair of sampling weeks within all three doses (all *p* < 0.001). At the week-3 endpoint, the blocked ANOVA showed significant differences among the four aquaponic groups (F_3,6_ = 14,330.43, *p* < 0.001), with the numerical order CA < CAS2 < CAS4 < CAS8; all six Tukey-adjusted pairwise comparisons were significant. Although the higher protein content observed in the aquaponic groups may be consistent with greater nutrient availability, the present experiment did not directly quantify nitrogen availability or assimilation. Therefore, differences in protein accumulation should be interpreted as observed dose-associated responses rather than direct evidence of enhanced nitrogen assimilation [8,14,15]. A more direct test would require measurements of nitrogen concentration in the aquaponic solution, plant nitrogen uptake, nitrogen-use efficiency, and, ideally, isotopic tracing.

**Figure 6:**
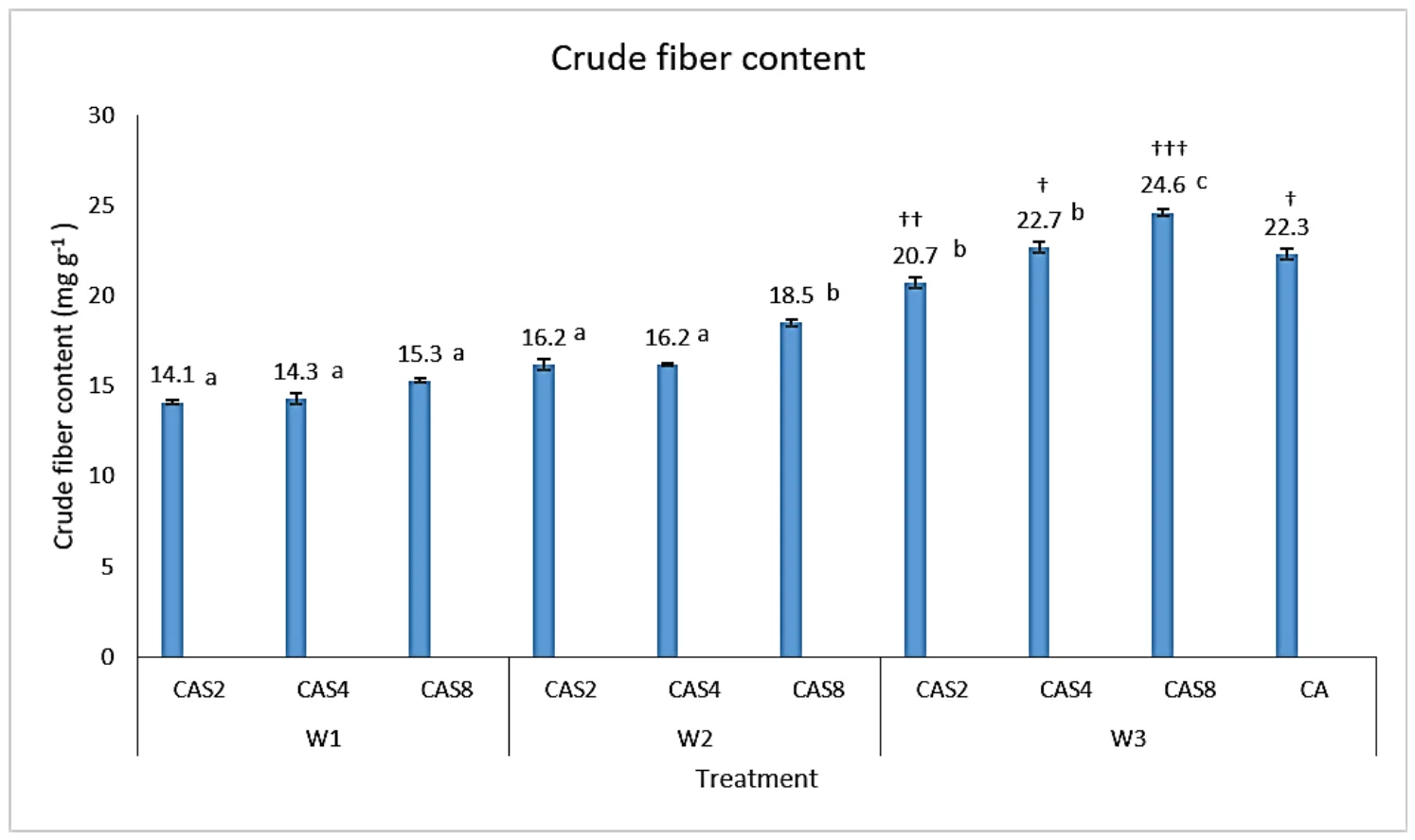
Crude fiber content of aquaponically cultivated celery following Spirulina supplementation. Crude fiber content was measured after one (W1), two (W2), and three (W3) weeks of supplementation with 2, 4, and 8 g L^−1^ of Spirulina. Values are presented as mean ± SD (n = 3 independent tank-level biological replicates). Different letters within each week indicate significant differences among Spirulina doses based on Tukey-adjusted simple-effect comparisons following a significant Spirulina dose × week interaction (*p* < 0.05). The longitudinal analysis only included CAS2, CAS4, and CAS8 because CA was sampled only at week 3. ^†^ *p* < 0.05; ^††^ *p* < 0.01; ^†††^ *p* < 0.001, based on the prespecified Dunnett comparisons between the Spirulina-supplemented treatments (CAS2, CAS4, and CAS8) and the untreated aquaponic control (CA) at week 3.

Crude fiber content also increased during cultivation, with the highest week-3 value recorded in CAS8 (24.6 ± 0.2 mg g^−1^ DW), followed by CAS4 (22.7 ± 0.3 mg g^−1^ DW), CA (22.3 ± 0.3 mg g^−1^ DW), and CAS2 (20.7 ± 0.3 mg g^−1^ DW) as shown in Table 4 and Figure 6. Compared with CA, CAS8 showed an approximately 10.8% higher crude fiber content at week 3. CA and CAS4 did not differ significantly, whereas CAS2 differed significantly from both CA and CAS4, and CAS8 differed significantly from CA, CAS2, and CAS4 (Tukey’s test, *p* < 0.05). Crude fiber showed significant Spirulina-dose, week, and Spirulina-dose × week effects. Because the interaction was significant, simple-effect comparisons were interpreted separately by week and dose. At W1, CAS2 did not differ significantly from CAS4 (Tukey-adjusted *p* = 0.754), whereas both were lower than CAS8 (*p* < 0.001). At W2, CAS2 and CAS4 again did not differ (*p* = 1.000), whereas both were lower than CAS8 (*p* < 0.001). By W3, all three Spirulina doses differed significantly from one another (all *p* < 0.001). Within each dose, crude fiber increased significantly across all three-week comparisons (*p* < 0.001). At the week-3 endpoint, the blocked ANOVA showed significant differences among the four aquaponic groups (F_3,6_ = 141.94, *p* < 0.001).

The higher crude fiber values may be associated with differences in plant growth and tissue development under the respective cultivation conditions. Previous studies have proposed that microalgal biostimulants may influence carbon assimilation, nutrient acquisition, and structural carbohydrate formation. However, cellulose, hemicellulose, photosynthetic carbon assimilation, and related cell-wall biosynthetic processes were not directly measured in the present study. These mechanisms should therefore be regarded as plausible hypotheses rather than demonstrated explanations for the observed fiber response [16,19].

**Figure 7:**
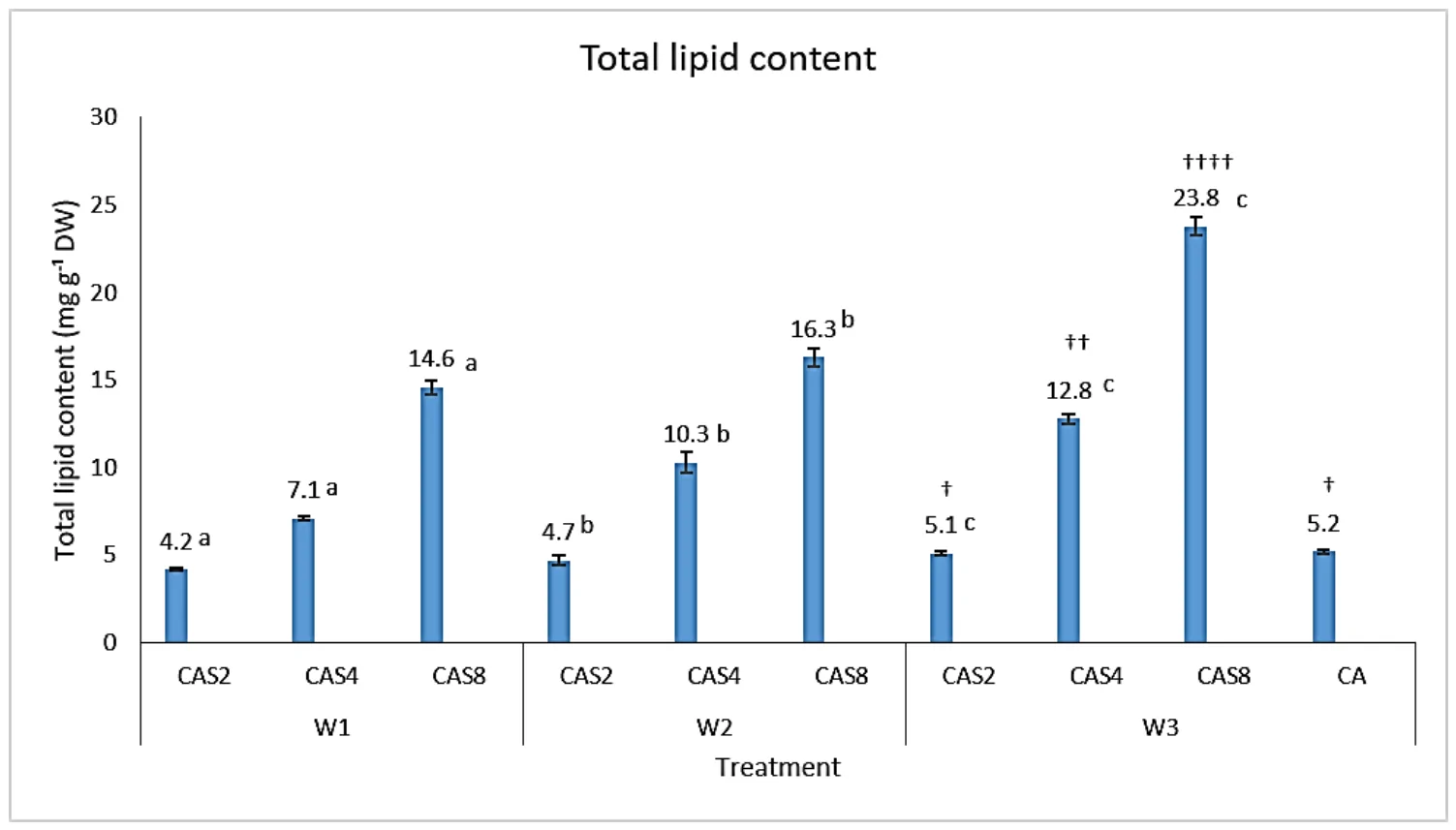
Lipid content of aquaponically cultivated celery following Spirulina supplementation. Lipid content was measured after one (W1), two (W2), and three (W3) weeks of supplementation with 2, 4, and 8 g L^−1^ Spirulina. Values are presented as mean ± SD (n = 3 independent tank-level biological replicates). Different letters within each week indicate significant differences among Spirulina doses based on Tukey-adjusted simple-effect comparisons following a significant Spirulina dose × week interaction (*p* < 0.05). The longitudinal analysis only included CAS2, CAS4, and CAS8 because CA was sampled only at week 3. ^†^ *p* < 0.05; ^††^ *p* < 0.01; ^††††^ *p* < 0.0001, based on the prespecified Dunnett comparisons between the Spirulina-supplemented treatments (CAS2, CAS4, and CAS8) and the untreated aquaponic control (CA) at week 3.

Total lipid content similarly showed higher values at higher supplementation levels and later sampling points (Table 5, Figure 7). At week 3, CAS8 showed the highest total lipid content (23.8 ± 0.5 mg g^−1^ DW), approximately 4.6-fold higher than CA (5.2 ± 0.1 mg g^−1^ DW) and 5.8-fold higher than CS (4.1 ± 0.1 mg g^−1^ DW). CAS4 reached 12.8 ± 0.3 mg g^−1^ DW. CA and CAS2 did not differ significantly, whereas CAS4 was significantly higher than CA and CAS2, and CAS8 was significantly higher than CA, CAS2, and CAS4 (Tukey’s test, *p* < 0.05). Total lipid content showed significant Spirulina-dose, week, and Spirulina-dose × week effects. Simple-effect analysis showed that CAS2, CAS4, and CAS8 differed significantly from one another within each sampling week. Temporal changes varied among doses: CAS2 showed no significant difference between W1 and W2 (Tukey-adjusted *p* = 0.275) but differed between W1 and W3 (*p* = 0.023), whereas CAS4 and CAS8 showed significant changes across the sampling period. At W3, CAS4 exceeded CAS2 by 7.76 mg g^−1^ DW (95% CI 7.00–8.52, *p* < 0.001), CAS8 exceeded CAS2 by 18.71 mg g^−1^ DW (95% CI 17.87–19.47, *p* < 0.001), and CAS8 exceeded CAS4 by 10.95 mg g^−1^ DW (95% CI 10.19–11.71, *p* < 0.001). At the week-3 endpoint, the blocked ANOVA showed significant differences among the four aquaponic groups (F_3,6_ = 2617.67, *p* < 0.001).

Total lipid content showed the strongest nutritional response at the higher Spirulina concentrations. The present results are consistent with the possibility that Spirulina supplementation affected primary carbon metabolism, but they do not demonstrate enhanced de novo lipid biosynthesis. The distinction is important because total lipid measurements do not identify individual fatty acids or determine whether changes resulted from altered fatty-acid synthesis, changes in membrane composition, differences in tissue development, or other metabolic processes. Recent experimental metabolomic work in lettuce and tomato showed that microalgal extracts can induce distinct changes in carbon utilization and storage depending on the crop and extract chemistry, providing a mechanistic context for interpreting the present lipid response without implying a specific pathway [8,22]. Targeted fatty-acid profiling, photosynthetic measurements, and measurements of soluble sugars and starch would be required to determine whether the observed increase in total lipid content reflects a broader reorganization of carbon metabolism.

Taken together, the protein, crude fiber, and lipid results indicate that Spirulina supplementation was associated with changes in the nutritional composition of celery, particularly at the higher application concentrations. The magnitude and direction of these responses varied among nutritional variables, indicating that Spirulina did not produce a uniform effect across all components. Further work combining targeted biochemical measurements with independent experiments would be required to determine the physiological mechanisms underlying these responses.

#### 2.3.2. Mineral Profiling

The mineral composition of celery varied according to the mineral element, Spirulina concentration, and sampling time.

Sodium concentrations increased substantially following Spirulina supplementation, particularly at higher application rates and longer cultivation periods (Table S1, Figure S1). At week 3, CAS8 showed the highest sodium concentration among the aquaponic treatments (339.9 ± 10.9 µg g^−1^ DW), compared with 153.0 ± 4.7 µg g^−1^ DW in untreated aquaponic celery (CA). CA and CAS2 did not differ significantly, whereas CAS4 was significantly higher than both CA and CAS2, and CAS8 was significantly higher than CA, CAS2, and CAS4 (Tukey’s test, *p* < 0.05). The longitudinal analysis showed significant differences among Spirulina-dose (F_3,6_ = 2040.75), week (F_3,6_ = 74.07), and Spirulina-dose × week interaction (F_3,6_ = 13.23, all *p* < 0.001). Simple-effect analysis showed significant differences among CAS2, CAS4, and CAS8 within each sampling week. Temporal changes differed among doses. CAS2 did not differ between W1 and W2 (*p* = 0.748) but showed significant differences between W1 and W3 (estimated difference = −22.52 µg g^−1^ DW, 95% CI −35.21 to −9.83, *p* < 0.001) and W2 and W3 (−18.92, 95% CI −31.61 to −6.23, *p* = 0.004). CAS4 differed significantly between W1 and W2 and between W1 and W3 but not between W2 and W3. CAS8 differed significantly between W1 and W3 and between W2 and W3. At W3, CAS4 exceeded CAS2 by 71.35 µg g^−1^ DW (95% CI 58.66–84.04, *p* < 0.001), while CAS8 exceeded CAS2 and CAS4 by 185.76 and 114.41 µg g^−1^ DW, respectively (both *p* < 0.001). The week-3 endpoint analysis also showed a significant treatment effect (F_3,6_ = 347.90, *p* < 0.001).

The higher sodium concentration is likely associated with both the mineral composition of Spirulina and the continuous recycling of dissolved nutrients within the aquaponic system. However, the present experiment did not directly quantify Na fluxes or the Na/K ratio, so these mechanisms remain tentative. The approximately threefold higher Na concentration at CAS8 than in CS, together with the lower and variable K concentrations observed across treatments, warrants attention to Na/K balance, osmotic regulation, and possible long-term salinity effects. Accordingly, 8 g L^−1^ should be regarded as the highest tested concentration rather than a recommended agronomic rate. Until longer-term studies establish mineral homeostasis, Na/K ratios, electrical conductivity responses, and plant tolerance, a practical upper limit for Spirulina application should not be generalized beyond the present experimental range [5,16,23].

Unlike the other minerals, potassium concentrations did not exhibit a consistent dose- or time-dependent increase following Spirulina supplementation (Table S1, Figure S1). Among the Spirulina-treated groups, values ranged from 57.2 ± 1.4 to 105.0 ± 2.2 µg g^−1^ DW, with the highest week-3 value observed in CAS4. At week 3, CAS2 and CAS4 did not differ significantly, CA differed significantly from both CAS2 and CAS4, and CAS8 differed significantly from CA, CAS2, and CAS4 (Tukey’s test, *p* < 0.05). Potassium showed significant Spirulina-dose (F_3,6_ = 209.24), week (F_3,6_ = 206.64), and Spirulina-dose × week interaction (F_3,6_ = 164.42, all *p* < 0.001) among CAS2, CAS4, and CAS8. Simple-effect analysis demonstrated a strong time-dependent dose response. At W1, all three doses differed significantly. At W2, all three doses again differed significantly, but the ranking changed. At W3, CAS2 and CAS4 did not differ significantly (Tukey-adjusted *p* = 0.677), whereas CAS8 differed significantly from both CAS2 and CAS4 (both *p* < 0.001). Within CAS2, W1 did not differ from W3 (*p* = 0.955), whereas W1 differed from W2 and W2 differed from W3 (both *p* < 0.001). CAS4 and CAS8 also showed significant temporal changes across most or all pair-wise comparisons. The week-3 endpoint analysis showed a significant treatment effect (F_3,6_ = 102.98, *p* < 0.001).

The variable potassium response may be influenced by interactions among potassium and other cations in the recirculating solution, as well as by differences in plant growth and tissue composition, including a possible dilution effect associated with the greater biomass observed in some Spirulina-treated groups. However, nutrient fluxes, ion competition, transporter activity, and whole-plant potassium content were not directly measured in the present study. These explanations should therefore be regarded as possible interpretations rather than demonstrated mechanisms [14,19].

Phosphorus accumulation increased progressively with increasing Spirulina concentration and cultivation duration (Table S1, Figure S1). The highest phosphorus concentration was observed in celery treated with CAS8 after three weeks (28.0 ± 0.4 µg g^−1^ DW), compared with 20.1 ± 0.2 µg g^−1^ DW in CA and 15.7 ± 0.3 µg g^−1^ DW in CS. At week 3, CA and CAS4 did not differ significantly. CAS2 differed significantly from both CA and CAS4, whereas CAS8 differed significantly from CA, CAS2, and CAS4 (Tukey’s test, *p* < 0.05). Phosphorus showed significant Spirulina-dose (F_3,6_ = 706.60), week (F_3,6_ = 2167.93), and Spirulina-dose × week interaction (F_3,6_ = 212.97, all *p* < 0.001). At W1, CAS2 did not differ significantly from CAS4 (Tukey-adjusted *p* = 0.095), whereas both differed from CAS8. At W2 and W3, all three doses differed significantly from one another. Temporal comparisons were significant for all week pairs within each dose (all *p* < 0.001). At W3, CAS4 exceeded CAS2 by 2.38 µg g^−1^ DW (95% CI 1.79–2.97, *p* < 0.001), CAS8 exceeded CAS2 by 9.93 µg g^−1^ DW (95% CI 9.34–10.52, *p* < 0.001), and CAS8 exceeded CAS4 by 7.55 µg g^−1^ DW (95% CI 6.96–8.14, *p* < 0.001). The week-3 endpoint analysis showed a significant treatment effect (F_3,6_ = 450.30, *p* < 0.001).

The enhanced phosphorus accumulation may be attributed to the phosphorus-rich composition of Spirulina together with improved phosphorus mobilization within the aquaponic system. Previous studies have suggested that microalgal biostimulants may enhance phosphorus acquisition by stimulating root growth, improving nutrient-use efficiency, and increasing phosphorus availability within the rhizosphere [8]. Since phosphorus is essential for ATP synthesis, nucleic acid metabolism, membrane phospholipid formation, and energy transfer, increased phosphorus availability may have contributed to the improved vegetative growth and biomass accumulation observed in Spirulina-treated celery [8,24]; however, these mechanisms were not directly investigated in the present study.

Calcium concentrations remained relatively stable during the first two weeks of cultivation but increased markedly following supplementation in CAS8 after three weeks (23.4 ± 2.4 µg g^−1^ DW), compared with 9.9 ± 0.3 µg g^−1^ DW in CA and 7.8 ± 0.4 µg g^−1^ DW in CS (Table S1, Figure S1). At week 3, CA, CAS2, and CAS4 did not differ significantly from one another, whereas CAS8 was significantly higher than CA, CAS2, and CAS4 (Tukey’s test, *p* < 0.05). Calcium showed significant Spirulina-dose effect (F_3,6_ = 101.32), week (F_3,6_ = 174.83) and Spirulina-dose × week interaction (F_3,6_ = 61.90, all *p* < 0.001) among CAS2, CAS4, and CAS8. No pairwise dose comparison was significant at W1. At W2, none of the three dose comparisons reached significance after Tukey adjustment. At W3, CAS8 was significantly higher than CAS2 (estimated difference = 13.49 µg g^−1^ DW, 95% CI 11.64–15.34, *p* < 0.001) and CAS4 (12.50, 95% CI 10.66–14.35, *p* < 0.001), whereas CAS2 and CAS4 did not differ (*p* = 0.375). Within CAS2, CAS4, and CAS8, the largest temporal differences occurred between W1/W3 and W2/W3, with the strongest response in CAS8. The week-3 endpoint analysis showed a significant treatment effect (F_3,6_ = 74.92, *p* < 0.001).

Calcium is indispensable for cell wall formation, membrane stability, intracellular signaling, and stress tolerance. The increased calcium accumulation observed in Spirulina-treated celery may reflect improved calcium acquisition and translocation associated with the biostimulatory effects of Spirulina on plant growth and nutrient utilization. Similar enhancements in calcium nutrition have been reported following application of microalgal biostimulants, which improve nutrient acquisition through stimulation of root growth and transporter activity. Improved calcium nutrition may also contribute to enhanced tissue firmness and postharvest quality [16].

At week 3, the blocked endpoint analysis was interpreted with the heteroscedasticity-robust sensitivity analysis. The conventional blocked ANOVA showed a treatment effect (F_3,6_ = 6.10, *p* = 0.0297), whereas the HC3 sensitivity analysis gave F_3,6_ = 3.24, *p* = 0.1028. Thus, the overall week-3 iron treatment effect was not statistically significant under the robust sensitivity analysis. The estimated treatment differences relative to CA were unchanged by the HC3 procedure: CAS2–CA = 0.0433 µg g^−1^ DW, CAS4–CA = 0.2600 µg g^−1^ DW, and CAS8–CA = 0.2667 µg g^−1^ DW. The conventional Tukey-adjusted comparisons were not treated as evidence of a statistically resolved week-3 treatment effect under the robust sensitivity analysis. Accordingly, the week-3 iron result is reported cautiously and is not interpreted as statistically significant.

Iron plays essential roles in chlorophyll biosynthesis, photosynthetic electron transport, respiration, and numerous redox enzymes. The increased iron concentration may partly reflect the relatively high iron content of Spirulina together with enhanced mineral accumulation induced by its biostimulant activity. Similar increases in iron accumulation following Spirulina supplementation have recently been reported in sustainable cropping systems and have been associated with improved micronutrient biofortification [16].

The mineral responses were strongly element-specific and changed over time. This element-specific behavior is important because it indicates that Spirulina supplementation did not simply increase the accumulation of all measured minerals in parallel. Sodium showed a strong dose-associated increase, whereas potassium showed a less consistent pattern; phosphorus and calcium showed substantial dose-associated responses, and week-3 iron required robust sensitivity analysis because of heteroscedasticity. The analysis confirms significant longitudinal dose × week interactions for Na, K, P and Ca, among CAS2, CAS4, and CAS8, while the week-3 iron treatment effect was not statistically significant under the HC3 sensitivity analysis. The mineral responses are compatible with the possibility that the applied *A. platensis* biomass contributed nutrients and/or modified nutrient availability within the aquaponic system. Published primary studies confirm that Spirulina biomass can contain appreciable amounts of the minerals examined in the present study. Čmiková et al. reported Ca, Mg, K, Na, and Fe concentrations of 2840, 4830, 14,400, 17,600, and 471 mg kg^−1^ dry biomass, respectively [25]. Ennaji et al. reported a phosphorus concentration of approximately 7335 mg kg^−1^ dry biomass [23]. These are literature-based nominal inputs rather than measured nutrient additions, and they do not account for the potential mineral content introduced with the biomass and do not indicate the fraction dissolved, retained in the aquaponic system, transformed, or taken up by plants These values illustrate that Spirulina biomass can represent a non-negligible source of mineral nutrients [8,19,26]. Therefore, the observed plant responses cannot be attributed exclusively to a biostimulant effect. Direct nutrient contribution from the applied *A. platensis* biomass and biostimulatory effects may both have contributed to the observed responses.

### 2.4. Analysis of Aflatoxins

No AFB1, AFB2, AFG1, AFG2, or AFM1 was detected above the analytical detection capability of the applied LC–MS/MS method in the tested celery samples.

The absence of detectable aflatoxins may partly reflect the controlled greenhouse environment, which can reduce exposure to environmental conditions favorable for fungal growth and mycotoxin production. However, because no fungal challenge or positive control was included, the present study cannot determine whether the absence of detectable aflatoxins was related to Spirulina supplementation or to the cultivation conditions [8,27].

### 2.5. GC-MS Chemical Profiling

GC–MS analysis revealed distinct differences in the relative metabolite profiles of soil-grown celery (CS), untreated aquaponically cultivated celery (CA), and Spirulina-treated aquaponic celery (CAS2, CAS4, and CAS8). The analysis was performed on representative samples without independent biological replication and was therefore used for qualitative and semi-quantitative descriptive profiling rather than inferential statistical testing. Compound assignments were based on comparison of acquired mass spectra with the NIST mass spectral library, characteristic mass-spectral fragmentation patterns, and comparison of experimental and literature retention indices (RI). Because authentic reference standards were not analyzed, all compounds were considered putatively identified, with agreement between experimental and literature RI values providing additional support for the proposed assignments.

The detected metabolites were grouped into seven major chemical classes: hydrocarbons, sesquiterpenes, monoterpenes and oxygenated monoterpenes, phthalides, diterpenes and diterpene alcohols, long-chain fatty alcohols, and phytosterols and antioxidant-associated metabolites (Table S2; Figures S2–S5). The profiles showed marked descriptive differences between soil-grown and aquaponically cultivated celery, together with additional variation among the Spirulina-treated aquaponic samples. Because the dataset was not independently replicated, these differences are presented as descriptive patterns rather than statistically supported Spirulina-dose effects.

The observed variation is consistent with the recognized plasticity of celery volatile and semi-volatile composition, which may vary according to plant organ, genotype, cultivation conditions, extraction procedure, and analytical platform. Previous GC–MS investigations have identified monoterpenes and phthalides as characteristic constituents of celery, while recent work on Egyptian aquaponically cultivated celery has also demonstrated substantial variation in volatile composition under aquaponic conditions. Accordingly, the metabolite profile observed in the present study should be regarded as characteristic of the investigated plant material and experimental conditions rather than as a universal chemical signature of soil-grown or aquaponically cultivated celery [3,26].

At the class level, soil-grown celery showed relatively greater representation of hydrocarbons and monoterpenes/oxygenated monoterpenes, whereas the aquaponically cultivated samples showed greater representation of phthalides, diterpenes and diterpene alcohols, and long-chain fatty alcohols. Within the aquaponic samples, the largest numerical difference associated with increasing Spirulina supplementation was observed for diterpene and diterpene-alcohol metabolites. Their combined relative peak-area contribution increased from 21.46% in CA to 26.04%, 29.52%, and 37.20% in CAS2, CAS4, and CAS8, respectively, compared with 0.49% in CS. A signal putatively assigned to neophytadiene represented a major component of the diterpene-associated relative peak area, accounting for 17.92% in CA and 21.39%, 25.46%, and 29.40% in CAS2, CAS4, and CAS8, respectively. Because neophytadiene may be formed as a chlorophyll/phytol-related thermal degradation product during GC analysis, these signals are not interpreted as confirmed endogenous neophytadiene metabolites or as evidence of biological accumulation. Phytol contributed a further 3.54% in CA and 4.65%, 4.06%, and 7.80% in CAS2, CAS4, and CAS8, respectively. These observations identify diterpene-associated metabolites as a prominent numerical feature of the analyzed samples; however, because the GC–MS analysis was not independently replicated, they should not be interpreted as a statistically validated concentration-response relationship [26,28].

Phthalides showed a different descriptive pattern. Total major phthalides accounted for 0.41% of the relative GC–MS peak area in CS and 14.53% in CA, whereas CAS2, CAS4, and CAS8 showed 9.07%, 14.37%, and 15.02%, respectively. Sedanolide was the predominant detected phthalide, contributing 12.13% in CA, 9.07% in CAS2, 11.70% in CAS4, and 11.24% in CAS8. Butylidenephthalide accounted for 1.07%, 1.47%, and 2.11% in CA, CAS4, and CAS8, respectively, while senkyunolide represented 1.33%, 1.20%, and 1.67%, respectively. Thus, the large difference in phthalide representation was primarily associated with the transition from soil-grown to aquaponically cultivated celery, whereas the Spirulina-treated samples showed a non-linear modulation of this profile. The phthalide-rich profile was largely maintained at the higher supplementation levels rather than displaying a simple linear concentration response. Phthalides are recognized characteristic constituents of celery and contribute substantially to its volatile profile [29].

Phytosterols and antioxidant-associated metabolites showed another descriptive pattern. These compounds were not detected in the CS or CA profiles but were detected in the Spirulina-treated samples, with combined relative peak-area representation of 3.87%, 9.84%, and 4.88% in CAS2, CAS4, and CAS8, respectively. The highest numerical representation occurred in CAS4. Stigmasterol contributed 3.87%, 4.34%, and 3.39% in CAS2, CAS4, and CAS8, respectively, while γ-sitosterol was particularly represented in CAS4 (4.34%) and α-tocopherol was detected at 1.16% in CAS4 and 1.06% in CAS8. This pattern differed from the diterpene-associated response, suggesting that the relative representation of these metabolite groups was not uniformly proportional to the applied Spirulina concentration. Nevertheless, their detection by GC–MS does not establish enhanced antioxidant capacity and bioavailability, and targeted quantitative and biological assays would be required to substantiate such implications [28,30].

Long-chain fatty alcohols showed an opposite descriptive pattern. Their combined relative representation increased from 1.38% in CS to 26.84% in CA and then decreased to 16.29%, 16.81%, and 14.96% in CAS2, CAS4, and CAS8, respectively. Similar changes were observed for the major individual constituents; n-tetracosanol, 1-heptacosanol, and octacosanol decreased from 7.20%, 8.24%, and 11.40% in CA to 3.72%, 4.51%, and 6.73%, respectively, in CAS8. These observations indicate that Spirulina supplementation was not associated with a generalized increase in all lipid-associated metabolite classes. Long-chain fatty alcohols may be related to cuticular wax and other lipid-associated processes and may respond to environmental and physiological conditions; however, the present data do not establish that Spirulina specifically suppressed wax biosynthesis because neither wax composition nor the relevant biosynthetic pathways was directly assessed [8,31,32].

The soil-grown samples also differed markedly in their hydrocarbon and monoterpene representation. Hydrocarbons accounted for 23.12% of the relative GC–MS peak area in CS, compared with 1.50% in CA and 1.75% in CAS8. Sesquiterpenes represented 3.51% in CS but were not represented in the aquaponic profiles, while monoterpenes and oxygenated monoterpenes accounted for 21.60% in CS and were not represented in the aquaponic profiles. The monoterpene-rich soil-grown profile was largely attributable to D-limonene (12.66%), eucalyptol (3.82%), α-pinene (2.80%), and camphene (1.23%). These findings further illustrate the strong influence of cultivation conditions on the relative composition of volatile and semi-volatile metabolites.

Taken together, the class-level results suggest two broad descriptive patterns. First, the transition from soil-grown to aquaponically cultivated celery was associated with a substantial restructuring of the relative GC–MS profile, with greater representation of phthalides, diterpene-associated metabolites, and long-chain fatty alcohols and lower representation of several hydrocarbon and monoterpene classes. Second, within the aquaponic background, Spirulina supplementation was associated with selective rather than uniform changes in relative metabolite representation. Diterpene and diterpene-alcohol metabolites showed the largest numerical increase toward CAS8, phytosterol- and antioxidant-associated metabolites showed their highest numerical representation at CAS4, and long-chain fatty alcohols showed lower relative representation following supplementation. These patterns are consistent with the recognized capacity of cultivation conditions and biostimulants to influence plant physiological and biochemical status, although the underlying mechanisms were not directly investigated in the present study [8,28,30].

Experimental metabolomic studies have similarly shown that microalgal-derived preparations can alter plant metabolite profiles, physiological and defense-associated biochemical responses across developmental stages, although the magnitude and nature of these responses depend strongly on environmental conditions and treatment mode [24,33]. However, the GC–MS findings should nevertheless be interpreted within the analytical limitations of the study. Because the GC–MS analysis was not independently replicated, the reported relative peak-area percentages cannot be used to estimate biological variability or statistical significance among treatments. Similarly, relative peak-area representation does not provide absolute metabolite concentrations and does not establish bioavailability, biological efficacy, or direct metabolic pathways. Confirmation of key metabolite identities using authentic reference standards, independent biological replication, and targeted quantitative analyses would strengthen these observations. Additional antioxidant, nutritional, sensory, bioavailability, and biological studies would be required to determine whether the descriptive metabolite differences translate into measurable functional effects [28,30].

### 2.6. Potential Practical and Sustainability Implications

From a commercial perspective, the higher relative peak-area representation of diterpenes, phthalides, phytosterols, and antioxidant-associated metabolites may improve its attractiveness as a functional vegetable for functional food applications. In particular, the simultaneous preservation of aroma-associated phthalides and valuable terpenoids and phytosterols provides a phytochemical profile that may be desirable for premium vegetable production [34].

The integration of Spirulina supplementation into aquaponic cultivation may also contribute to sustainable agricultural production. Microalgal biostimulants provide a renewable source of bioactive compounds that can improve crop quality while potentially reducing dependence on synthetic agricultural inputs. Combined with the inherent advantages of aquaponic systems, including efficient water utilization, nutrient recycling, and reduced environmental impact, Spirulina supplementation represents a promising strategy for producing high-value medicinal and aromatic vegetables. Nevertheless, comprehensive economic analyses, including production costs, yield response, and market acceptance, are required to determine the commercial feasibility of large-scale implementation [35,36].

However, the present study did not evaluate production costs, input requirements, economic returns, consumer acceptance, sensory attributes, or market value. Therefore, the potential of *A. platensis*-supplemented celery as a value-added or functional-food product should be considered preliminary and require further investigation. Future studies should include techno-economic and cost–benefit analyses comparing *A. platensis* supplementation with conventional nutrient inputs, together with sensory and consumer-acceptance studies. Such assessments would help determine whether the observed biological and nutritional benefits can translate into economically and commercially viable production while potentially reducing reliance on synthetic inputs.

## 3. Materials and Methods

### 3.1. Plant Material

Celery (*Apium graveolens* L.) seedlings were obtained from SEKEM Organization, Egypt and cultivated according to the aquaponic production protocol described by Abouelela et al. (2025) [3]. Seedlings were transplanted 21 days after sowing when they reached approximately 5.0 ± 0.3 cm in height and had developed 3–5 true leaves.

### 3.2. Experimental Design

The aquaponic experiment was independently repeated three times under the same experimental conditions. In each experimental repetition, four independent tanks were used, with one tank assigned to each aquaponic group: untreated aquaponic celery (CA), and celery supplemented with *A. platensis* at 2, 4, and 8 g L^−1^ (CAS2, CAS4, and CAS8, respectively). Forty celery plants were cultivated in each tank. The tank was considered the experimental unit and independent biological replicate, whereas individual plants within each tank were considered subsamples and were not treated as independent biological replicates. Thus, each aquaponic treatment was represented by three independent tank-level biological replicates (n = 3) across the three independent experimental repetitions; the three experimental repetitions were not multiplied to give n = 9.

For growth evaluation, at the beginning of each independent experimental repetition, one plant was randomly selected from each treatment tank and individually identified. The same plant was followed and measured at weeks 1, 2, and 3, allowing leaf length and width to be analyzed as repeated observations using plant identity as a random effect. For destructive measurements, different plants were harvested at each sampling week. Plants remaining in the tanks were maintained until the subsequent sampling point, so W1, W2, and W3 destructive measurements did not represent repeated measurements of the same individual plants.

**Figure 8:**
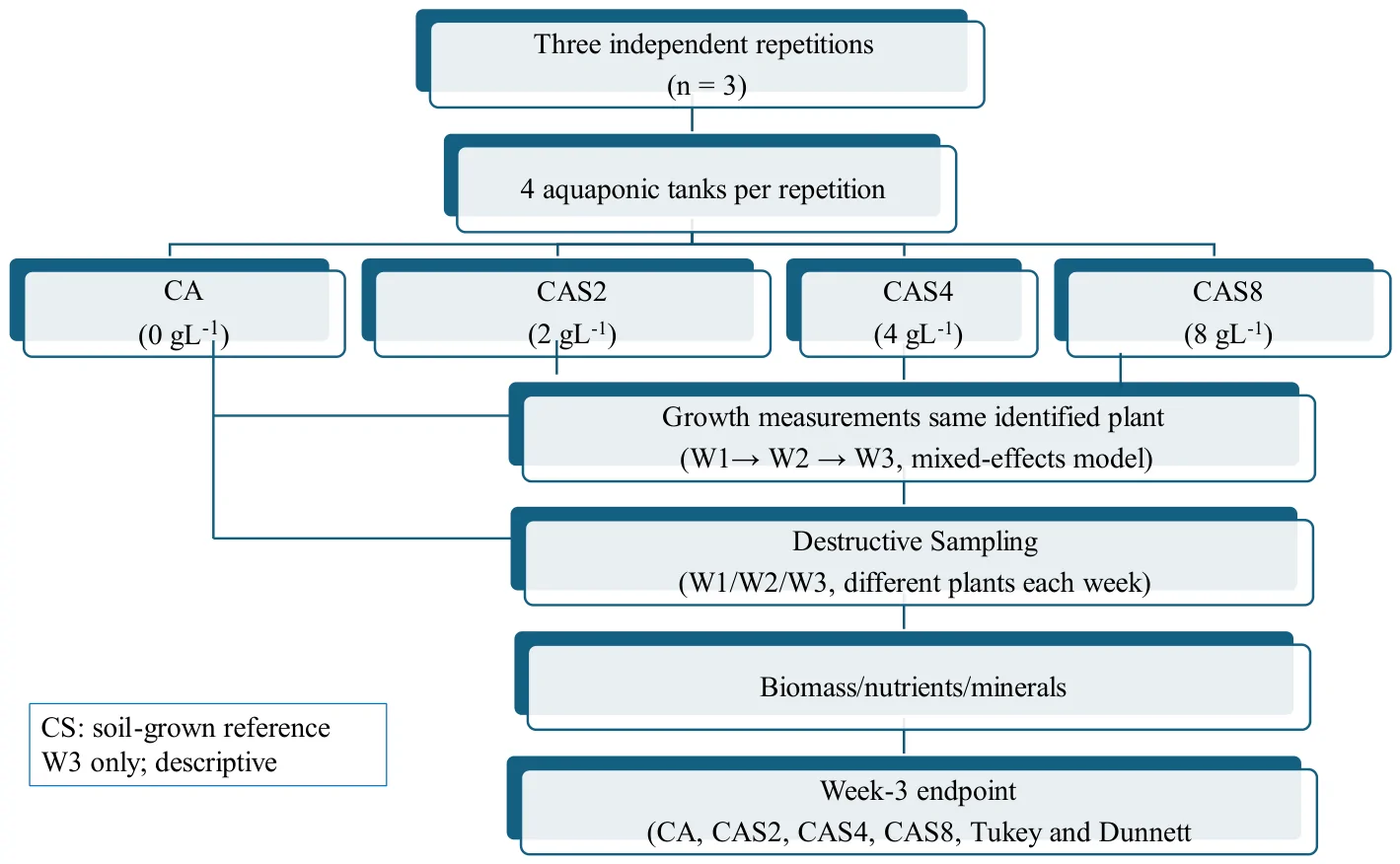
Schematic overview of the experimental design and sampling workflow. The aquaponic experiment comprised three independent experimental repetitions, with four treatment tanks per repetition: untreated aquaponic celery (CA) and celery supplemented with 2, 4, or 8 g L^−1^ of *Arthrospira platensis* (CAS2, CAS4, and CAS8). The tank was the experimental unit and biological replicate (n = 3 per treatment). Growth measurements were obtained repeatedly from the same individually identified plant at W1, W2, and W3, whereas destructive biomass, nutritional, and mineral measurements were obtained from different plants at each sampling week. CA was sampled only at W3 and therefore was included only in the separate week-3 endpoint analysis. Soil-grown celery (CS) was sampled at W3 as a descriptive reference.

Untreated aquaponic celery (CA) and soil-grown celery (CS) were sampled only at week 3. Accordingly, CA was used as the untreated aquaponic control for the separate week-3 endpoint analyses, whereas CS was retained as a descriptive week-3 reference because it was cultivated under different environmental and management conditions. The complete experimental workflow is summarized schematically in Figure 8.

### 3.3. Soil and Aquaponic Cultivation

**Figure 9:**
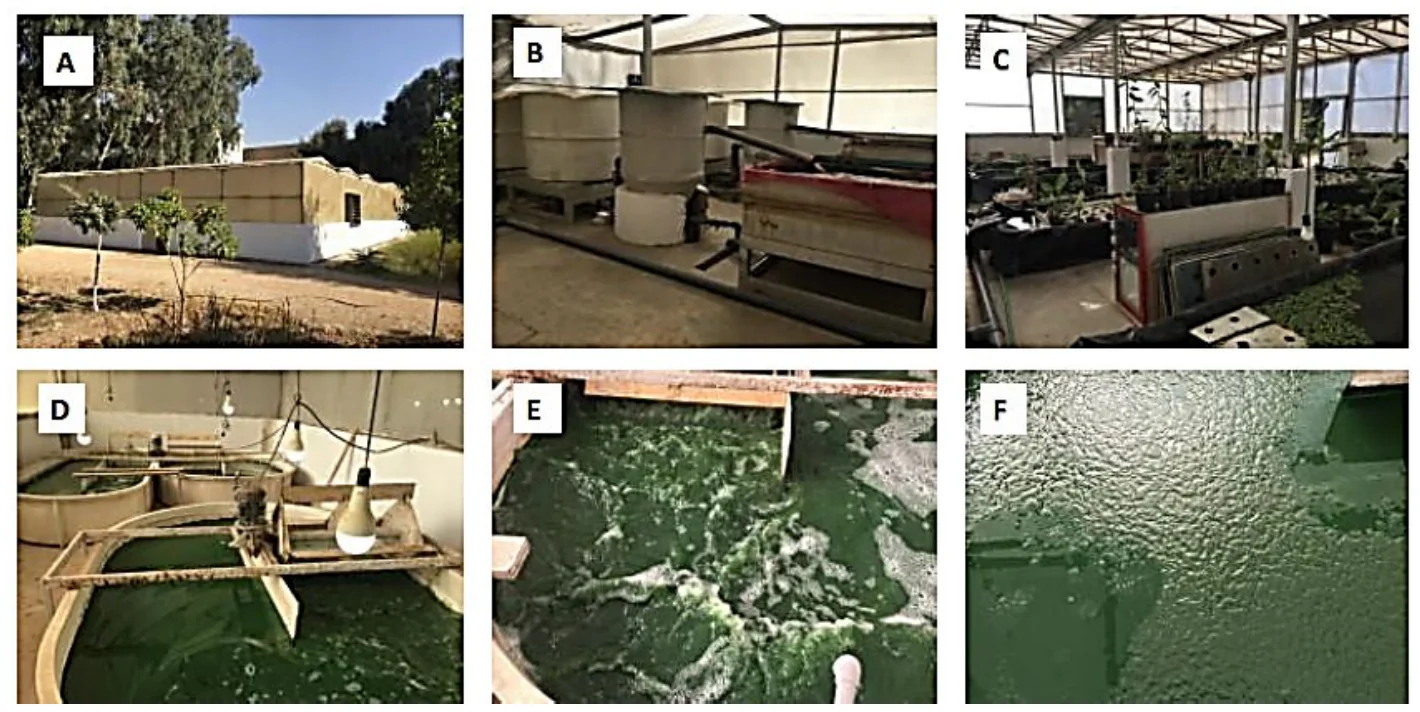
Experimental aquaponic system used for celery cultivation. (**A**) Greenhouse facility at Heliopolis University for Sustainable Development; (**B**) recirculating aquaculture system (RAS) including fish tanks and filtration units; (**C**) deep-water culture (DWC) hydroponic beds used for celery cultivation; (**D**) Spirulina cultivation unit; (**E**) Spirulina culture during agitation; (**F**) floating Spirulina biomass at the water surface prior to harvesting.

The experiment was conducted between January and March 2025 at the aquaponic research facility of Heliopolis University for Sustainable Development, Cairo, Egypt, according to the cultivation procedures described by Abouelela et al. (2025) and Siringi (2025) [3,37]. Celery seedlings were cultivated either under conventional organic soil conditions or in an integrated aquaponic production system (Figure 9). For soil cultivation, the experimental field had previously been amended with organic manure and managed according to certified organic farming practices. Weeds were removed manually throughout the cultivation period, and plants were irrigated daily using a drip irrigation system.

### 3.4. Aquaponic System

The aquaponic experiment was conducted inside a polyethylene-covered greenhouse (50 × 28 m) located at the Botanical Garden of Heliopolis University (Figure 9). The greenhouse consisted of two interconnected sections: an insulated aquaculture unit (25 × 14 m) and an adjacent plant cultivation area. The integrated aquaponic system combined a recirculating aquaculture system (RAS) with a deep-water culture (DWC) hydroponic unit. The RAS consisted of four fish culture tanks (1500 L each), a swirl separator, a 100-µm static screen filter, a trickling biofilter, and a sump tank connected through a continuous recirculation system using a pump. Solid waste collected by sedimentation and filtration was transferred to an aerated remineralization tank, and the nutrient-rich supernatant was periodically returned to the aquaponic system to enhance nutrient recycling. The hydroponic component consisted of four DWC tanks, each providing a cultivation area of approximately 8 m^2^ and accommodating 40 plants, resulting in a total cultivation area of 32 m^2^ and a total cultivation capacity of 160 plants. The four DWC tanks were operated as separate treatment units, with each tank assigned exclusively to one aquaponic treatment. Spirulina was added only to the designated treatment tank, and treatment water was not intentionally transferred between tanks. Water temperature was maintained at 24–27 °C, dissolved oxygen was above 5.0 mg L^−1^, pH was 6.5–7.2, electrical conductivity was 1.5 mS cm^−1^, and total ammonia nitrogen (TAN) was below 1.5 mg L^−1^ throughout the experiment. System operation and management followed the procedures described by Abouelela et al. (2025), as summarized in Table 6 [3].

### 3.5. Fish Stocking and Management

A total of 1200 Nile tilapia (*Oreochromis niloticus*) juveniles (300 fish per tank) with an initial body weight of 20.0–25.0 g and total length of 10–13 cm were stocked into the aquaculture system at an initial stocking density of 200 fish m^−3^, corresponding to an initial biomass of 4.0–5.0 kg m^−3^. Fish were randomly distributed among tanks to ensure uniform biomass. Fish were fed a commercial pelleted diet containing 28–30% crude protein at 2–3% of body weight per day, divided into two equal meals. Feeding rates were adjusted periodically according to fish growth. The greenhouse operated under a natural photoperiod (approximately 12–13 h light), with an average air temperature of 25 ± 3 °C and relative humidity of 60–70%.

### 3.6. Preparation and Application of Spirulina

Spirulina biomass was produced by the Heliopolis University Spirulina Production Unit (Figure 9). Dried Spirulina biomass was suspended in distilled water immediately before application, thoroughly homogenized, and introduced directly into the recirculating nutrient solution to obtain final concentrations of 2, 4, or 8 g L^−1^. Untreated aquaponic plants received no Spirulina supplementation and served as the aquaponic control. Applications were performed once weekly for three consecutive weeks. The stated concentrations represent the nominal concentrations immediately following each weekly application. The Spirulina concentration in the recirculating nutrient solution was not continuously monitored between applications; therefore, persistence of nominal concentrations throughout each weekly interval was not assumed.

### 3.7. Growth Evaluation

Plant growth was evaluated weekly for three weeks after treatment initiation. At the beginning of each independent experimental repetition, one plant was randomly selected from each treatment tank and individually identified. The same plants were followed throughout the three-week experiment, and maximum leaf length and width were measured at weeks 1, 2, and 3 using a digital ruler. Three fully expanded leaves were selected from each plant for measurement.

### 3.8. Determination of Dry Matter

Shoots and roots were harvested separately and initially shade-dried before oven drying at 65 °C for 72 h until constant weight. Dry matter content was calculated as:

Dry matter (%) = (Dry weight/Fresh weight) × 100

### 3.9. Determination of Total Protein

Total protein content was determined using the micro-Kjeldahl method following the Official Methods of Analysis of AOAC International [38]. For each determination, 0.5 g of freshly harvested plant material was weighed and subjected to Kjeldahl digestion with concentrated sulfuric acid in the presence of a catalyst mixture (potassium sulfate and copper sulfate). The digest was then alkalized with sodium hydroxide, followed by steam distillation of ammonia, which was collected in boric acid solution. The distillate was titrated against standardized hydrochloric acid to quantify total nitrogen. The nitrogen content was calculated from the titration volume after correction for the reagent blank. Protein content was obtained by multiplying the measured nitrogen content by a nitrogen-to-protein conversion factor of 6.25, based on the assumption that proteins contain approximately 16% nitrogen. The resulting protein concentration was normalized to the analyzed fresh sample mass and expressed as µg g^−1^ FW. The calculation pathway was:

Protein (%) = [(V × N × 14.007)/m] × F

where V is the volume of titrant consumed (mL), N is the normality of the hydrochloric acid titrant, 14.007 is the atomic mass of nitrogen (mg mmol^−1^), m is the fresh sample mass (mg), and F is the nitrogen-to-protein conversion factor (6.25). Protein concentrations were subsequently expressed on a fresh-weight basis as µg g^−1^ FW. Samples designated for protein determination were analyzed immediately after harvesting.

### 3.10. Determination of Crude Fiber

Crude fiber content was determined using the filter bag method according to AOAC International (2013) [38]. Plant material designated for crude fiber analysis was oven-dried and subsequently ground to a fine powder before analysis.

### 3.11. Determination of Total Lipids

Total lipid content was determined using a rapid Soxhlet extraction method according to AOAC International (2013) [38]. Plant material designated for lipid analysis was oven-dried and subsequently ground to a fine powder. Samples were first hydrolyzed with boiling hydrochloric acid to release bound lipids. After filtration and drying, lipids were extracted with petroleum ether. Following solvent evaporation, lipid residues were dried to constant weight, and total lipid content was expressed as percentage (*w*/*w*).

### 3.12. Mineral Analysis

The concentrations of sodium (Na), potassium (K), calcium (Ca), phosphorus (P), and iron (Fe) were determined by inductively coupled plasma-atomic emission spectroscopy (ICP–AES; Thermo Scientific iCAP 6000 Series, Thermo Fisher Scientific, Cambridge, United Kingdom) following microwave-assisted acid digestion according to AOAC International (2013) [38]. Plant material designated for mineral analysis was oven-dried and ground to a fine powder before analysis.

### 3.13. Aflatoxin Analysis

Aflatoxins were analyzed by liquid chromatography–tandem mass spectrometry (LC–MS/MS) following the multi-mycotoxin UPLC–MS/MS method described by Dreolin et al. [39]. Plant material designated for aflatoxin analysis was oven-dried and ground to a fine powder before analytical processing. The targeted aflatoxins included AFB1, AFB2, AFG1, AFG2, and AFM1. The published method reported an instrument limit of detection of 0.0003 ng mL^−1^ for these aflatoxins and method limits of quantification as low as 0.1 µg kg^−1^ in the validated cereal-based matrix. These published sensitivity values were used only as methodological context because matrix-specific LOD/LOQ values were not independently established for the celery samples analyzed in the present study.

### 3.14. Gas Chromatography-Mass Spectrometry (GC-MS)

GC–MS analysis was performed at the Center for Drug Discovery Research and Development, Faculty of Pharmacy, Ain Shams University, Cairo, Egypt, following the analytical protocol reported by Abouelela et al. (2025) [3]. Representative samples were collected separately for GC–MS profiling and processed according to the GC–MS analytical protocol. The analysis was performed on representative samples without independent biological replication and therefore, the resulting metabolite data were used for qualitative and semi-quantitative descriptive profiling and were not subjected to inferential statistical testing. The detected compounds were putatively identified by comparison of their acquired mass spectra with reference spectra in the NIST mass spectral library, taking into consideration the characteristic mass spectral fragmentation patterns and the agreement between the experimentally determined retention indices (RI) and corresponding literature RI values. The experimental RI values showed generally close agreement with the reported literature values, providing additional support for the proposed compound identities. As no authentic reference standards were analyzed, none of the detected compounds were considered confirmed by authentic-standard comparison. Therefore, all compound assignments are reported as putative identifications [40]. Relative metabolite abundance was calculated from integrated GC peak areas and expressed as percentages of the total ion chromatogram (TIC) peak area (% peak area).

### 3.15. Statistical Analysis

The tank was considered the experimental unit for the aquaponic treatments, whereas individual plants within a tank were considered subsamples. Thus, each Spirulina dose was represented by three independent tank-level biological replicates (n = 3) across the three experimental repetitions. The three experimental repetitions were treated as independent biological replicates and were not treated as nine independent replicates. The soil-grown control (CS) was maintained separately from the aquaponic system and was replicated using three independent pots (n = 3). Because CS plants were sampled only at week 3 and were cultivated under different environmental and management conditions from the aquaponic treatments, CS was used as a descriptive week-3 reference and was not included in the longitudinal aquaponic analyses.

All statistical analyses were performed using R (version 4.3.2) within RStudio (Release 2026.09.0). Linear mixed-effects models were fitted using the lme4 package (version 1.1–35; function lmer) with restricted maximum likelihood (REML). For mixed-effects models, fixed-effect tests used the Satterthwaite approximation for denominator degrees of freedom as implemented by the lmerTest package (version 3.1-3). Because the final models for the destructive measurements were ordinary linear models with experimental repetition specified as a fixed blocking factor, ML and REML estimation were not applicable to those analyses. Likelihood-ratio χ^2^ tests were not used; fixed effects in the mixed-effects models were evaluated using F-tests with Satterthwaite-adjusted denominator degrees of freedom, whereas the ordinary linear models were evaluated using the conventional model F-tests and corresponding F-distribution *p*-values. Estimated marginal means and pairwise contrasts for the longitudinal mixed-effects and linear models were obtained using the emmeans package (version 1.10.3), with Tukey adjustment applied to the relevant families of pairwise comparisons. For the week-3 blocked one-way ANOVA, Tukey’s HSD procedure was implemented using the base R stats package (R version 4.3.2) (functions aov and TukeyHSD) for pairwise comparisons among CA, CAS2, CAS4, and CAS8. Prespecified comparisons of CAS2, CAS4, and CAS8 against CA were implemented using the multcomp package (version 1.4-25; function glht) with Dunnett adjustment. For the week-3 blocked comparisons, the residual degrees of freedom were 6. Post-hoc results are reported as estimated mean differences with their standard errors, 95% confidence intervals, and multiplicity-adjusted *p*-values. Random-effect variance estimates, convergence diagnostics, and singularity checks were reported for the final mixed-effects models.

Growth measurements. For each experimental repetition, one plant was randomly selected from each Spirulina-dose tank at the beginning of the experiment and individually identified. The same plant was then followed throughout the experiment, and leaf length and leaf width were measured at weeks 1, 2, and 3. Thus, the three measurements obtained from each selected plant represented repeated observations from the same individual and were not treated as independent observations. Leaf length and width were analyzed using linear mixed-effects models with treatment, week, and treatment × week interaction as fixed effects and individual plant identity as a random intercept. Experimental repetition was not included as a separate random effect because only three independent experimental repetitions were available; the tank was retained as the experimental unit, while plant identity was used to account for within-plant repeated measurements.

For each response variable (leaf length and leaf width), the model was specified as:

Y ~ treatment × week + (1 | plant)

where Y represents the response variable, treatment represents the aquaponic treatment group, week represents sampling week (W1–W3), and plant represents the individually identified plant. The plant-level random intercept was included to account for the correlation among repeated measurements from the same plant.

Both models were fitted using REML. The estimated plant-level random-intercept variance was 0.02333 for leaf length and 0.02500 for leaf width. Both models converged successfully and were not classified as singular according to lme4::isSingular using a tolerance of 1 × 10^−4^. These variance estimates indicate that the random plant effect was non-zero and therefore supported retaining plant identity in the repeated-measures growth models.

At the week-3 endpoint, the four aquaponic groups (CA, CAS2, CAS4, and CAS8) were additionally compared using a blocked one-way analysis of variance with experimental repetition as a fixed blocking factor. The model was specified as:

Y ~ treatment + repetition

with residual degrees of freedom equal to 6. Treatment was tested using the blocked ANOVA F-test, with experimental repetition included to account for systematic differences among the three independent experimental repetitions. Tukey-adjusted pairwise comparisons were used for all four-group comparisons, and Dunnett-adjusted comparisons were used for the prespecified comparisons of CAS2, CAS4, and CAS8 against CA. CS was retained only as a descriptive week-3 reference.

When a significant treatment × week interaction was detected, simple-effect comparisons were performed using estimated marginal means and pairwise contrasts implemented with the emmeans package. Two sets of simple-effect comparisons were evaluated: (i) pairwise comparisons among the Spirulina doses (CAS2, CAS4, and CAS8) within each sampling week (W1, W2, and W3), and (ii) pairwise comparisons among sampling weeks (W1, W2, and W3) within each Spirulina dose. Tukey adjustment was applied to control multiplicity within each set of pairwise comparisons. Simple-effect results were reported as estimated mean differences, standard errors, 95% confidence intervals, and Tukey-adjusted *p*-values. These comparisons were used as follow-up analyses to interpret significant treatment × week interactions and did not replace the primary longitudinal mixed-effects models.

Destructive biochemical, nutritional, and biomass measurements. For destructive measurements, different plants were harvested at each sampling week. Therefore, measurements from W1, W2, and W3 were not repeated measurements of the same individual plant. Plants harvested from the same tank were treated as subsamples, and the tank-level analytical result represented the biological replicate.

Because CA was sampled only at week 3, longitudinal analyses of destructive measurements were restricted to the three Spirulina-supplemented groups (CAS2, CAS4, and CAS8). For each longitudinal destructive response variable, the model was specified as:

Y ~ treatment × week + repetition

When a significant treatment × week interaction was detected, simple-effect comparisons were performed to identify the source of the interaction. Two sets of comparisons were evaluated: (i) pairwise comparisons among Spirulina doses (CAS2, CAS4, and CAS8) within each sampling week (W1, W2, and W3), and (ii) pairwise comparisons among sampling weeks (W1, W2, and W3) within each Spirulina dose. Tukey adjustment was applied to control multiplicity within each set of pairwise comparisons. Simple-effect results were reported as estimated mean differences, standard errors, 95% confidence intervals, and multiplicity-adjusted *p*-values. These comparisons were treated as follow-up analyses of significant treatment × week interactions and were not used to replace the primary longitudinal model.

Treatment is represented as CAS2, CAS4, and CAS8; week is represented as W1–W3; and experimental repetition was included as a fixed blocking factor. These models were fitted as ordinary linear models using the lm function in R. Because no random-effects term was included, maximum likelihood (ML) and restricted maximum likelihood (REML) estimation were not applicable. Fixed-effect significance was assessed using the model F-tests and corresponding F-distribution *p*-values; likelihood-ratio χ^2^ tests were not used. The treatment term in these longitudinal analyses therefore represents the overall Spirulina-dose effect among CAS2, CAS4, and CAS8, while the treatment × week term represents the Spirulina-dose × week interaction among the supplemented groups. CA was not included in these longitudinal models because it was not sampled at W1 or W2. CA was included in the separate week-3 endpoint analysis. For the longitudinal destructive models, experimental repetition had three levels and was therefore treated as a fixed blocking factor rather than a random effect. Preliminary mixed-effects models treating repetition as a random effect produced boundary or near-zero random-effect variance estimates for several response variables. Given the small number of experimental repetitions (three), the fixed-block specification was retained for the final analyses to avoid unstable estimation of a random-effect variance.

For week-3 comparisons, CA, CAS2, CAS4, and CAS8 were analyzed using a blocked one-way analysis of variance with experimental repetition as the fixed blocking factor. The model was specified as:

Y ~ treatment + repetition

with residual degrees of freedom equal to 6. The treatment effect was evaluated using the blocked ANOVA F-test. Tukey-adjusted pairwise comparisons were calculated using Tukey’s HSD procedure, and 95% confidence intervals for estimated pairwise differences were reported. Dunnett-adjusted comparisons were used for the prespecified comparisons of CAS2, CAS4, and CAS8 versus CA.

When a significant treatment × week interaction was detected for a longitudinal destructive response, simple-effect comparisons were performed using estimated marginal means and pairwise contrasts with the emmeans package. Pairwise comparisons among CAS2, CAS4, and CAS8 were performed separately within W1, W2, and W3, and pairwise comparisons among W1, W2, and W3 were performed separately within each Spirulina dose. Tukey adjustment was applied to the pairwise comparisons within each comparison family. Estimated mean differences, standard errors, 95% confidence intervals, and Tukey-adjusted *p*-values were reported. Because CA was sampled only at week 3, it was not included in these longitudinal simple-effect comparisons.

Model diagnostics. Model assumptions were evaluated using residual-versus-fitted plots, normal Q–Q plots, studentized residuals, and influence diagnostics. Shapiro–Wilk tests were used as supplementary diagnostics for residual normality, and nonconstant variance was assessed using residual-based variance diagnostics. Because only three independent biological replicates were available per treatment, formal assumption tests were interpreted cautiously and were considered together with graphical diagnostics rather than being used as the sole basis for model acceptance or rejection. The growth mixed-effects models converged successfully and showed positive plant-level random-effect variances without singularity. For leaf length, the estimated plant-level random-intercept variance was 0.02333; for leaf width, it was 0.02500. No singular fit was detected for either model. For the destructive measurements, preliminary models that included experimental repetition as a random effect produced boundary or near-zero variance estimates for several response variables. Because experimental repetition had only three independent levels, repetition was therefore modeled as a fixed blocking factor in the final destructive analyses. This approach avoids relying on an unstable random-effect variance estimate based on only three levels while retaining the blocking structure of the experimental design. Evidence of heteroscedasticity was observed for fresh biomass, and dry biomass in the longitudinal analyses and for week-3 iron. These responses were therefore evaluated using heteroscedasticity-robust (HC3) sensitivity inference. Dry matter was not subjected to HC3 sensitivity analysis because its fitted model showed essentially zero residual variance and was therefore excluded from formal inferential analysis. No observation was excluded solely on the basis of statistical influence because no experimental or analytical justification for exclusion was identified. The HC3 results were used as a sensitivity analysis rather than as a replacement for the prespecified model structure, and the corresponding findings were interpreted cautiously given the limited number of independent biological replicates. No observation was excluded solely on the basis of statistical influence because no experimental or analytical justification for exclusion was identified.

Multiple comparisons and reporting. For longitudinal models with a significant treatment × week interaction, estimated marginal means and pairwise contrasts were obtained using the emmeans package. Tukey adjustment was used for pairwise comparisons among CAS2, CAS4, and CAS8 within each sampling week and for pairwise comparisons among W1, W2, and W3 within each Spirulina dose. Estimated mean differences, standard errors, 95% confidence intervals, and Tukey-adjusted *p*-values were reported for these simple-effect comparisons.

For the separate week-3 blocked one-way ANOVA, Tukey adjustment was used for the complete set of pairwise comparisons among CA, CAS2, CAS4, and CAS8. In addition, Dunnett adjustment was used for the three prespecified treatment-versus-control comparisons (CAS2 vs. CA, CAS4 vs. CA, and CAS8 vs. CA). Thus, Tukey adjustment was used for the broader pairwise characterization of treatment differences, whereas Dunnett adjustment was reserved for the prespecified comparisons of each Spirulina treatment against the CA control. Multiplicity was controlled within each prespecified comparison family rather than by applying a single adjustment across all analyses. All post-hoc comparisons are reported as estimated mean differences with their standard errors, 95% confidence intervals, and multiplicity-adjusted *p*-values. Statistical significance was defined as *p* < 0.05.

GC–MS analysis. GC–MS metabolite profiling was not independently replicated and was therefore excluded from inferential statistical analysis. GC–MS results are presented as relative peak-area percentages and were interpreted descriptively to characterize differences in metabolite occurrence and relative representation among the analyzed samples. Accordingly, no *p*-values, standard deviations, or statistical significance statements were assigned to individual GC–MS metabolites or metabolite classes.

Data from the independent biological replicates are presented as mean ± standard deviation (SD; n = 3).

## 4. Conclusions

The present study showed that Spirulina supplementation was associated with dose- and time-related differences in growth, biomass, nutritional composition, and mineral accumulation among the supplemented aquaponic celery groups. Significant Spirulina dose × week interactions for several responses indicated that the magnitude of dose-associated differences varied across cultivation stages and therefore required interpretation through simple-effect comparisons. By week 3, the 8 g L^−1^ treatment generally showed the highest growth and biomass and the highest values for protein, phosphorus, calcium, and several other measured responses, whereas potassium showed a non-monotonic response and sodium increased markedly at higher supplementation levels.

Because the untreated aquaponic control (CA) was sampled only at week 3, the longitudinal analyses were restricted to CAS2, CAS4, and CAS8 and therefore describe differences among Spirulina doses over time rather than a longitudinal comparison between Spirulina supplementation and the untreated control. Comparisons involving CA were restricted to week-3 endpoint analyses, while CS was used only as a contextual week-3 reference. These findings indicate that Spirulina supplementation was associated with measurable changes in celery performance under the conditions of the present experiment; however, the magnitude and generalizability of these responses should be interpreted in light of the limited number of independent biological replicates and the specific experimental conditions.

Model diagnostics supported the overall adequacy of most statistical analyses, with residual-versus-fitted and normal Q–Q plots, influence diagnostics, and mixed-model convergence and singularity assessments incorporated into the revised analysis. Selected outcomes showed evidence of nonconstant residual variance and were therefore examined using heteroscedasticity-robust sensitivity analyses. The HC3 sensitivity analysis did not change the conclusions for fresh or dry biomass, whereas it changed the week-3 iron endpoint from significant under the conventional blocked ANOVA to not statistically significant under HC3. Dry matter was interpreted descriptively because its inferential model exhibited essentially zero residual variance.

The GC–MS analysis provided descriptive evidence of differences in the relative representation of detected metabolite classes among the analyzed samples. Because these measurements were obtained from representative samples without independent biological replication, and because the aquaponic and soil-grown plants were cultivated under different environmental and management conditions, the GC–MS results should not be interpreted as evidence that Spirulina supplementation or cultivation system caused or determined the observed metabolite differences.

Future studies with greater biological replication, targeted quantitative metabolite profiling using authentic standards, and direct measurements of nutrient inputs, uptake, and mass balance will be valuable for distinguishing direct nutrient contributions of Spirulina from physiological biostimulant effects. Additional work incorporating molecular or transcriptomic approaches, bioavailability and sensory assessment, and techno-economic evaluation would further clarify the mechanisms and practical feasibility of Spirulina-assisted aquaponic celery production.

## Figures and Tables

**Table 1:** Temporal changes in leaf length and width in aquaponically cultivated celery among the Spirulina-supplemented groups (CAS2, CAS4, and CAS8).

Treatment	Leaf Length (cm) ± SD	Leaf Width (cm) ± SD
CS, W3	2.0 ± 0.1	2.1 ± 0.1
CA, W3	2.3 ± 0.3 ^†^	2.5 ± 0.2 ^†^
CAS2, W1	1.2 ± 0.1 ^a^	1.7 ± 0.2 ^a^
CAS2, W2	1.9 ± 0.2 ^a^	2.6 ± 0.1 ^a^
CAS2, W3	2.5 ± 0.1 ^a†^	2.7 ± 0.1 ^a††^
CAS4, W1	1.9 ± 0.2 ^b^	2.2 ± 0.1 ^b^
CAS4, W2	2.4 ± 0.3 ^b^	3.0 ± 0.3 ^b^
CAS4, W3	3.8 ± 0.1 ^b††^	3.9 ± 0.2 ^b†††^
CAS8, W1	2.5 ± 0.1 ^c^	2.5 ± 0.1 ^b^
CAS8, W2	2.6 ± 0.2 ^b^	3.1 ± 0.2 ^b^
CAS8, W3	4.6 ± 0.2 ^c†††^	4.8 ± 0.2 ^c††††^

CS, soil-grown celery; CA, untreated aquaponic celery; CAS2–CAS8, aquaponic celery supplemented with Spirulina at 2, 4, or 8 g L^−1^; W1–W3, weeks 1–3 after supplementation. Values are presented as mean ± SD (n = 3 independent tank-level biological replicates). Growth measurements were analyzed longitudinally using a linear mixed-effects model with individual plant identity as a random intercept. Different lowercase letters within the same sampling week indicate significant differences among CAS2, CAS4, and CAS8 according to Tukey-adjusted simple-effect comparisons (*p* < 0.05). ^†^ *p* < 0.05; ^††^ *p* < 0.01; ^†††^ *p* < 0.001; ^††††^ *p* < 0.0001, based on the prespecified Dunnett comparisons between the Spirulina-supplemented treatments (CAS2, CAS4, and CAS8) and the untreated aquaponic control (CA) at week 3. CS was included only as a descriptive week-3 reference.

**Table 2:** Temporal changes in fresh biomass, dry biomass, and dry matter content in aquaponically cultivated celery among the Spirulina-supplemented groups (CAS2, CAS4, and CAS8).

Treatment	Fresh Weight (g) ± SD	Dry Weight (g) ± SD	Dry Matter (%)
CS, W3	173.0 ± 3.6	38.0 ± 0.7	22.0
CA, W3	110.1 ± 5.5 ^†^	19.5 ± 1.1 ^††^	17.7
CAS2, W1	10.5 ± 0.2 ^a^	0.7 ± 0.1 ^a^	6.7
CAS2, W2	65.5 ± 2.3 ^a^	7.8 ± 0.3 ^a^	11.9
CAS2, W3	110.6 ± 8.0 ^a†^	16.0 ± 1.1 ^a†^	14.5
CAS4, W1	20.9 ± 0.7 ^a^	2.1 ± 0.1 ^a^	10.0
CAS4, W2	95.4 ± 4.8 ^b^	13.3 ± 0.7 ^b^	13.9
CAS4, W3	215.6 ± 10.8 ^b††^	34.2 ± 1.7 ^b†††^	15.9
CAS8, W1	48.3 ± 2.4 ^b^	6.2 ± 0.4 ^b^	12.8
CAS8, W2	185.2 ± 9.3 ^c^	28.0 ± 1.4 ^c^	15.1
CAS8, W3	260.4 ± 13.0 ^c†††^	49.9 ± 2.0 ^c††††^	19.2

CS, soil-grown celery; CA, untreated aquaponic celery; CAS2–CAS8, aquaponic celery supplemented with Spirulina at 2, 4, or 8 g L^−1^; W1–W3, weeks 1–3 after supplementation. Values are presented as mean ± SD (n = 3 independent tank-level biological replicates). Different plants were harvested at each sampling week; therefore, observations across weeks were not repeated measurements of the same individual plants. Longitudinal fresh- and dry-biomass analyses used a fixed-block linear model with experimental repetition as the blocking factor, followed by Tukey-adjusted simple-effect comparisons when the Spirulina dose × week interaction was significant. Different lowercase letters within the same sampling week indicate significant differences among CAS2, CAS4, and CAS8 according to Tukey-adjusted simple-effect comparisons (*p* < 0.05). ^†^ *p* < 0.05; ^††^ *p* < 0.01; ^†††^ *p* < 0.001; ^††††^ *p* < 0.0001, based on the prespecified Dunnett comparisons between the Spirulina-supplemented treatments (CAS2, CAS4, and CAS8) and the untreated aquaponic control (CA) at week 3. CS was included only as a descriptive week-3 reference. Dry-matter values are presented descriptively because the longitudinal model showed essentially zero residual variance and was not used for formal inferential interpretation.

**Table 3:** Temporal changes in total protein content in aquaponically cultivated celery among the Spirulina-supplemented groups (CAS2, CAS4, and CAS8).

Treatment	Protein Content (µg g^−1^ FW) ± SD
CS, W3	92.2 ± 2.7
CA, W3	209.6 ± 4.3 ^†^
CAS2, W1	98.4 ± 1.7 ^a^
CAS2, W2	184.0 ± 3.3 ^a^
CAS2, W3	226.6 ± 4.3 ^a††^
CAS4, W1	116.7 ± 1.8 ^b^
CAS4, W2	242.5 ± 3.4 ^b^
CAS4, W3	271.7 ± 3.0 ^b†††^
CAS8, W1	137.3 ± 2.7 ^c^
CAS8, W2	325.3 ± 5.7 ^c^
CAS8, W3	376.2 ± 4.0 ^c††††^

CS, soil-grown celery; CA, untreated aquaponic celery; CAS2–CAS8, aquaponic celery supplemented with Spirulina at 2, 4, or 8 g L^−1^. Total protein was measured after one (W1), two (W2), and three (W3) weeks of supplementation with 2, 4, and 8 g L^−1^ of Spirulina. Different plants were harvested at each sampling week. Values are presented as mean ± SD (n = 3 independent tank-level biological replicates). For destructive measurements, different plants were harvested at each sampling week. Longitudinal analyses only included CAS2, CAS4, and CAS8 because CA was sampled only at week 3. Therefore, the Spirulina-dose and Spirulina-dose × week terms represent dose effects and dose × week interactions among the supplemented groups. Experimental repetition was modeled as a fixed blocking factor. Different superscript lowercase letters within the same sampling week indicate significant differences among CAS2, CAS4, and CAS8 according to Tukey-adjusted pairwise comparisons of estimated marginal means (*p* < 0.05), ^†^ *p* < 0.05; ^††^ *p* < 0.01; ^†††^ *p* < 0.001; ^††††^ *p* < 0.0001, based on the prespecified Dunnett comparisons between the Spirulina-supplemented treatments (CAS2, CAS4, and CAS8) and the untreated aquaponic control (CA) at week 3. CS was included only as a descriptive week-3 reference.

**Table 4:** Temporal changes in crude fiber content in aquaponically cultivated celery among the Spirulina-supplemented groups (CAS2, CAS4, and CAS8).

Treatment	Crude Fiber Content (mg g^−1^ DW) ± S.D.
CS, W3	10.4 ± 0.2
CA, W3	22.3 ± 0.3 ^†^
CAS2, W1	14.1 ± 0.1 ^a^
CAS2, W2	16.2 ± 0.3 ^a^
CAS2, W3	20.7 ± 0.3 ^a††^
CAS4, W1	14.3 ± 0.3 ^a^
CAS4, W2	16.2 ± 0.1 ^a^
CAS4, W3	22.7 ± 0.3 ^b†^
CAS8, W1	15.3 ± 0.1 ^b^
CAS8, W2	18.5 ± 0.2 ^b^
CAS8, W3	24.6 ± 0.2 ^c†††^

CS, soil-grown celery; CA, untreated aquaponic celery; CAS2–CAS8, aquaponic celery supplemented with Spirulina at 2, 4, or 8 g L^−1^. Crude fiber was measured after one (W1), two (W2), and three (W3) weeks of supplementation with 2, 4, and 8 g L^−1^ of Spirulina. Different plants were harvested at each sampling week. Values are presented as mean ± SD (n = 3 independent tank-level biological replicates). For destructive measurements, different plants were harvested at each sampling week. Longitudinal analyses only included CAS2, CAS4, and CAS8 because CA was sampled only at week 3. Therefore, the Spirulina-dose and Spirulina-dose × week terms represent dose effects and dose × week interactions among the supplemented groups. Experimental repetition was modeled as a fixed blocking factor. Different superscript lowercase letters within the same sampling week indicate significant differences among CAS2, CAS4, and CAS8 according to Tukey-adjusted pairwise comparisons of estimated marginal means (*p* < 0.05). ^†^ *p* < 0.05; ^††^ *p* < 0.01; ^†††^ *p* < 0.001, based on the prespecified Dunnett comparisons between the Spirulina-supplemented treatments (CAS2, CAS4, and CAS8) and the untreated aquaponic control (CA) at week 3. CS was included only as a descriptive week-3 reference.

**Table 5:** Temporal changes in total lipid content in aquaponically cultivated celery among the Spirulina-supplemented groups (CAS2, CAS4, and CAS8).

Treatment	Total Lipid Content (mg g^−1^ DW) ± SD
CS, W3	4.1 ± 0.1
CA, W3	5.2 ± 0.1 ^†^
CAS2, W1	4.2 ± 0.1 ^a^
CAS2, W2	4.7 ± 0.3 ^a^
CAS2, W3	5.1 ± 0.1 ^a†^
CAS4, W1	7.1 ± 0.1 ^b^
CAS4, W2	10.3 ± 0.6 ^b^
CAS4, W3	12.8 ± 0.3 ^b††^
CAS8, W1	14.6 ± 0.4 ^c^
CAS8, W2	16.3 ± 0.5 ^c^
CAS8, W3	23.8 ± 0.5 ^c††††^

CS, soil-grown celery; CA, untreated aquaponic celery; CAS2–CAS8, aquaponic celery supplemented with Spirulina at 2, 4, or 8 g L^−1^. Total lipid content was measured after one (W1), two (W2), and three (W3) weeks of supplementation with 2, 4, and 8 g L^−1^ of Spirulina. Different plants were harvested at each sampling week. Values are presented as mean ± SD (n = 3 independent tank-level biological replicates). For destructive measurements, different plants were harvested at each sampling week. Longitudinal analyses only included CAS2, CAS4, and CAS8 because CA was sampled only at week 3. Therefore, the Spirulina-dose and Spirulina-dose × week terms represent dose effects and dose × week interactions among the supplemented groups. Experimental repetition was modeled as a fixed blocking factor. Different superscript lowercase letters within the same sampling week indicate significant differences among CAS2, CAS4, and CAS8 according to Tukey-adjusted pairwise comparisons of estimated marginal means (*p* < 0.05). ^†^ *p* < 0.05; ^††^ *p* < 0.01; ^††††^ *p* < 0.0001, based on the prespecified Dunnett comparisons between the Spirulina-supplemented treatments (CAS2, CAS4, and CAS8) and the untreated aquaponic control (CA) at week 3. CS was included only as a descriptive week-3 reference.

**Table 6:** Experimental conditions and characteristics of the aquaponic cultivation system used for celery production.

Parameter	Description
Experimental site	Aquaponic Research Facility, Heliopolis University for Sustainable Development, Cairo, Egypt
Greenhouse	Polyethylene-covered greenhouse (Figure 9A)
Greenhouse dimensions	50 × 28 m
Aquaculture unit	Indoor recirculating aquaculture system (RAS) (Figure 9B)
Plant production system	Deep-water culture (DWC) hydroponic system (Figure 9C)
Fish species	Oreochromis niloticus (Nile tilapia)
Initial fish weight	20.0–25.0 g
Initial fish length	10.0–13.0 cm
Commercial feed	28–30% crude protein
Feeding rate	2–3% of fish biomass day^−1^ (two equal meals)
Experimental treatments	Soil-grown celery (CS), untreated aquaponic celery (CA), aquaponic celery supplemented with 2 (CAS2), 4 (CAS4), and 8 g L^−1^ (CAS8) of *Arthrospira platensis* (Spirulina)
Number of plants per treatment	40 plants per aquaponic tank; one tank per aquaponic treatment in each experimental repetition
Replicates used for analyses	Three independent tank-level biological replicates per treatment (n = 3), from three independent experimental repetitions
Seedling age at transplanting	21 days
Seedling height	5.0 ± 0.3 cm
Seedling developmental stage	3–5 leaves
Spirulina source	Spirulina Production Unit, Heliopolis University
Spirulina preparation	Powder suspended in distilled water immediately before application
Spirulina concentrations	0, 2, 4 and 8 g L^−1^
Application method	Added directly to the recirculating aquaponic nutrient solution
Application frequency	Once weekly for three consecutive weeks
Plant growth evaluation	Weekly for three weeks after Spirulina application (Figure 2)
Harvest evaluation	Final shoot and root morphology recorded at harvest (Figure 2)

## Data Availability

The data presented in this study are available from the corresponding author upon request.

## Supplementary Materials

The following supporting information can be downloaded at https://www.mdpi.com/article/10.3390/plants15192985/s1, Figure S1. Mineral content of aquaponically cultivated celery following Spirulina supplementation. Na, K, Ca, Fe, and P were measured after one (W1), two (W2), and three (W3) weeks of supplementation with 2, 4, and 8 g L^−1^ of Spirulina. Values are presented as mean ± SD (n = 3 independent tank-level biological replicates). Different letters within each week indicate significant differences among Spirulina doses based on Tukey-adjusted simple-effect comparisons following a significant Spirulina dose × week interaction (*p* < 0.05 The longitudinal analysis only included CAS2, CAS4, and CAS8 because CA was sampled only at week 3. Figure S2. Representative GC–MS chromatograms of metabolites detected in soil-grown celery (CS), untreated aquaponic celery (CA), and Spirulina-treated aquaponic celery (CAS2, CAS4, and CAS8). The chromatograms are presented for descriptive chemical profiling only; because the GC–MS analysis was not independently biologically replicated, no inferential statistical analysis was performed. Compound assignments were considered putative based on mass-spectral library matching and retention-index agreement and were not confirmed using authentic reference standards. Figure S3. Heatmap showing relative abundance of individual metabolites identified by GC–MS in soil-grown celery (CS), untreated aquaponic celery (CA), and Spirulina-treated aquaponic celery (CAS2, CAS4, and CAS8). The heatmap represents descriptive relative peak-area patterns; no inferential statistical analysis was performed on the GC–MS dataset. GC–MS results are descriptive only. Figure S4. Distribution of major metabolite classes identified by GC–MS in soil-grown, untreated aquaponic, and Spirulina-treated aquaponic celery. Values represent descriptive relative GC–MS peak-area percentages from the analyzed samples. No inferential statistical analysis was performed on the GC–MS dataset. Figure S5. GC–MS metabolite-class response profile of soil-grown and aquaponically cultivated celery and the observed differences associated with Spirulina supplementation. The profile is descriptive and does not represent statistical treatment effects. No inferential statistical analysis was performed on the GC–MS dataset. Table S1. Temporal changes in mineral content in aquaponically cultivated celery among the Spirulina-supplemented groups (CAS2, CAS4, and CAS8). Table S2. GC–MS profile of hexane extract of soil-grown celery (CS), untreated aquaponic celery (CA), and Spirulina-treated aquaponic celery (CAS2, CAS4, and CAS8). GC–MS measurements were obtained from representative samples and were not independently replicated; values therefore represent relative peak-area percentages and are presented descriptively. Class totals were calculated by summing the relative peak-area percentages of the individual compounds assigned to each predefined class; duplicate detections of the same compound were summed before class-level aggregation. Compound identities are putative based on NIST-library matching and retention-index agreement; no individual compound should be interpreted as an authentic-standard-confirmed identification.
