# Supplementary material for: Spirulina (Arthrospira platensis) Influences Nutritional Characteristics and the Relative GC–MS Chemical Profile of Aquaponically Cultivated Celery (Apium graveolens)
Source: Plants (Basel). 2026 Sep 30;15(19):2985. doi: 10.3390/plants15192985 (PMC13666756; doi:10.3390/plants15192985)
Supplement: Supplementary file 1 [file plants-15-02985-s001.zip › plants-4530137-supplementary.pdf]

Table S1. Temporal changes in mineral content in aquaponically cultivated celery among the Spirulina-supplemented groups (CAS2, CAS4, and CAS8).

Treatment	Na (μg g^{-1} DW, mean ± SD)	K (μg g^{-1} DW, mean ± SD)	P (μg g^{-1} DW, mean ± SD)	Ca (μg g^{-1} DW, mean ± SD)	Fe (μg g^{-1} DW, mean ± SD)
CS, W 3	110.0 ± 4.0	115.0 ± 3.3	15.7 ± 0.3	7.8 ± 0.4	0.7 ± 0.1
CA, W 3	153.0 ± 4.7 †	89.9 ± 2.3 ††	20.1 ± 0.2 †	9.9 ± 0.3 †	0.8 ± 0.2
CAS2, W 1	131.7 ± 4.0 ^{a}	104 ± 4.9 ^{a}	12.7 ± 0.1 ^{a}	7.4 ± 0.3 ^{a}	0.3 ± 0.1 ^{a}
CAS2, W 2	135.3 ± 4.4 ^{a}	73.7 ± 2.2 ^{a}	15.6 ± 0.3 ^{a}	7.9 ± 0.2 ^{a}	0.6 ± 0.1 ^{ab}
CAS2, W 3	154.2 ± 5.0 ^{a†}	103.0 ± 1.5 ^{a†}	18.0 ± 0.2 ^{a††}	9.9 ± 0.2 ^{a†}	0.8 ± 0.2 ^{b}
CAS4, W 1	176.5 ± 4.2 ^{b}	59.6 ± 2.5 ^{b}	13.2 ± 0.3 ^{a}	7.5 ± 0.4 ^{a}	0.3 ± 0.1 ^{a}
CAS4, W 2	223.4 ± 6.6 ^{b}	94.7 ± 2.7 ^{b}	16.5 ± 0.2 ^{b}	8.0 ± 0.1 ^{a}	0.8 ± 0.2 ^{a}
CAS4, W 3	225.5 ± 7.4 ^{b††}	105.0 ± 2.2 ^{a†}	20.4 ± 0.4 ^{c†}	10.9 ± 0.2 ^{a†}	1.0 ± 0.1 ^{a}
CAS8, W 1	307.9 ± 3.9 ^{c}	74.1 ± 1.8 ^{c}	14.9 ± 0.3 ^{b}	8.0 ± 0.1 ^{a}	0.6 ± 0.1 ^{a}
CAS8, W 2	312.3 ± 3.6 ^{c}	57.2 ± 1.4 ^{c}	17.7 ± 0.5 ^{c}	9.7 ± 0.4 ^{a}	0.7 ± 0.1 ^{a}
CAS8, W 3	339.9 ± 10.9 ^{c†††}	81.9 ± 1.4 ^{b†††}	28.0 ± 0.4 ^{c†††}	23.4 ± 2.4 ^{b††}	1.0 ± 0.1 ^{a}

CS, soil-grown celery; CA, untreated aquaponic celery; CAS2–CAS8, aquaponic celery supplemented with Spirulina at 2, 4, or 8 g L^{-1}.

Mineral content was measured after one (W1), two (W2), and three (W3) weeks of supplementation with 2, 4, and 8 g L^{-1} Spirulina. Different plants were harvested at each sampling week.

Values are presented as mean ± SD (n = 3 independent tank-level biological replicates). For destructive measurements, different plants were harvested at each sampling week. Longitudinal analyses included CAS2, CAS4, and CAS8 only because CA was sampled only at week 3. Therefore, the Spirulina-dose and Spirulina-dose × week terms represent dose effects and dose × week interactions among the supplemented groups. Experimental repetition was modeled as a fixed blocking factor. Different superscript lowercase letters within the same sampling week indicate significant differences among CAS2, CAS4, and CAS8 according to Tukey-adjusted pairwise comparisons of estimated marginal means ($p < 0.05$). † $p < 0.05$; †† $p < 0.01$; ††† $p < 0.001$, based on the prespecified Dunnett comparisons between the Spirulina-supplemented treatments (CAS2, CAS4, and CAS8) and the untreated aquaponic control (CA) at week 3. CS was included only as a descriptive week-3 reference.

Table S2. GC–MS profile of hexane extract of soil-grown celery (CS), untreated aquaponic celery (CA), and Spirulina-treated aquaponic celery (CAS2, CAS4, and CAS8). GC–MS measurements were obtained from representative samples and were not independently replicated; values therefore represent relative peak-area percentages and are presented descriptively. Class totals were calculated by summing the relative peak-area percentages of the individual compounds assigned to each predefined class; duplicate detections of the same compound were summed before class-level aggregation. Compound identities are putative based on NIST-library matching and retention-index agreement; no individual compound should be interpreted as an authentic-standard-confirmed identification.

No.	Rt (min.)	RI (exp.)	RI (lit.)	Compound name	Molecular formula	Base peak	Fragmentation pattern	CA	CAS2	CAS4	CAS8	CS
1	3.410-3.418	775-776	777	2-Hexanone	$C_6H_{12}O$	43	100, 85, 71, 58, 43, 41, 36	0.92	6.30	1.43	0.79	1.30
2	3.571	782	789	*trans*-1,3-Dimethyl-cyclohexane	C_8H_{16}	97	112, 98, 97, 70, 56, 55, 41, 35	0.92	-	2.25	1.13	3.41
3	3.655	785	780	3-Hexanol	$C_6H_{14}O$	59	101, 84, 73, 59, 55, 41, 35	0.29	-	0.46	-	0.72
4	3.735	788	780	2-Hexanol	$C_6H_{14}O$	45	101, 87, 70, 69, 45, 41, 35	0.43	-	-	0.27	-
5	4.022	799	806	Octane	C_8H_{18}	43	114, 98, 85, 71, 57, 43, 41, 37	0.58	-	1.31	0.62	8.19
6	4.638	823	830	Ethylcyclohexane	C_8H_{16}	55	112, 111, 84, 83, 67, 55, 41, 36	-	-	-	-	0.15
7	6.474	894	906	Nonane	C_9H_{20}	43	128, 99, 85, 71, 57, 43, 41	-	-	-	-	0.13
8	6.940	910	914	Tricyclene	$C_{10}H_{16}$	93	126, 121, 105, 93, 41	-	-	-	-	0.12
9	7.105	915	920	Sabinene	$C_{10}H_{16}$	93	136, 105, 93, 77, 65, 41	-	-	-	-	0.08
10	7.278	921	928	α-Pinene	$C_{10}H_{16}$	93	136, 121, 105, 93, 77, 53, 41	-	-	-	-	1.28
11	7.632	932	934	Camphene	$C_{10}H_{16}$	93	136, 121, 107, 93, 79, 67, 53, 41	-	-	-	-	1.23
12	7.875	940	948	3,3-Dimethyl-2-hexanone	$C_8H_{16}O$	43	120, 105, 85, 43	-	-	-	-	0.22
13	8.355	955	960	β-Pinene	$C_{10}H_{16}$	93	136, 121, 105, 93, 77, 53, 41	-	-	-	-	0.12

14	8.447	958	961	α-Pinene	$C_{10}H_{16}$	93	136, 121, 105, 93, 77, 53, 41	-	-	-	-	1.52
15	8.960	974	980	β-Myrcene	$C_{10}H_{16}$	93	136, 121, 93, 79, 69, 53, 41	-	-	-	-	0.38
16	9.521	992	1001	Undecane	$C_{11}H_{24}$	57	113, 99, 85, 71, 57, 43, 41, 36	-	-	0.28	0.14	-
17	9.557	994	996	1,8-Cineole (Eucalyptol)	$C_{10}H_{18}O$	43	154, 125, 111, 71, 69, 43, 41	-	-	-	-	1.88
18	9.702	998	998	γ-Terpinene	$C_{10}H_{16}$	121	136, 121, 105, 93, 77, 65, 41	-	-	-	-	0.44
19	9.799	1001	1005	p-Cymene	$C_{10}H_{14}$	119	134, 119, 103, 91, 65, 51	-	-	-	-	1.09
20	9.998	1008	1009	Eucalyptol	$C_{10}H_{18}O$	43	154, 139, 125, 108, 84, 81, 69, 55, 43, 41	-	-	-	-	1.94
21	10.107	1011	1018	D-Limonene	$C_{10}H_{16}$	68	136, 121, 107, 93, 79, 68, 53, 41	-	-	-	-	12.66
22	10.984	1038	1039	γ-Terpinene	$C_{10}H_{16}$	93	136, 121, 105, 93, 77, 65, 41	-	-	-	-	0.75
23	11.513	1055	1054	2-Propylhept-3-enoic acid, phenylthio ester	$C_{16}H_{22}OS$	69	218, 153, 125, 83, 69, 55, 41	-	-	-	-	0.33
24	11.915	1067	1069	2-Carene	$C_{10}H_{16}$	93	136, 121, 105, 93, 79, 67, 41	-	-	-	-	0.24
25	12.182	1076	1070	(1α,2β,4α)- 1,4-dimethyl-2-(2-methylpropyl)-cyclohexane	$C_{12}H_{24}$	69	167, 125, 111, 83, 69, 55, 41	-	-	-	-	0.21
26	12.645	1090	1095	*trans*-1-Ethyl-1,3-dimethyl-cyclohexane	$C_{10}H_{20}$	69	125, 111, 83, 69, 55, 41	-	-	-	-	0.29
27	12.685	1091	1110	Undecane	$C_{11}H_{24}$	57	113, 98, 85, 71, 57, 43, 41, 35	-	-	0.49	0.26	-

28	12.776	1094	1097	1,3-Dimethyladamantane	$C_{12}H_{20}$	149	164, 149, 121, 107, 93, 79, 67, 41	-	-	-	-	5.30
29	13.089	1104	1102	(−)-Camphor	$C_{10}H_{16}O$	95	152, 125, 108, 95, 81, 69, 55, 41	-	-	-	-	0.50
30	13.195	1107	1101	1-Isothiocyanato-3,5-dimethyl- adamantane	$C_{13}H_{19}NS$	163	163, 135, 121, 107, 93, 79, 69, 41	-	-	-	-	0.26
31	13.365	1113	1112	2,4-Diethyl-1-methyl-cyclohexane	$C_{11}H_{22}$	69	125, 95, 83, 69, 55, 41	-	-	-	-	0.23
32	13.555	1119	1118	*cis*-Menthone	$C_{10}H_{18}O$	69	139, 125, 112, 95, 83, 69, 55, 41	-	-	-	-	0.33
33	13.675	1123	1117	5,9-Tridecadien-1-ol, 10-propyl-, acetate	$C_{18}H_{32}O_2$	69	164, 125, 109, 93, 81, 69, 55, 41	-	-	-	-	0.28
34	13.805	1127	1130	3-Ethyl-5-methyl-1-propyl-cyclohexane	$C_{12}H_{24}$	69	168, 153, 139, 125, 97, 83, 69, 55, 41	-	-	-	-	0.42
35	14.074	1136	1139	1-Methyl-2-pentyl-cyclohexane	$C_{12}H_{24}$	97	168, 153, 139, 125, 97, 69, 55, 41	-	-	-	-	0.28
36	14.195	1140	1045	*cis*-1-Ethyl-1,4-dimethyl-cyclohexane	$C_{10}H_{20}$	69	135, 111, 97, 83, 69, 55, 41	-	-	-	-	0.27
37	14.365	1145	1154	Menthol	$C_{10}H_{20}O$	71	138, 123, 95, 81, 71, 69, 55, 41	-	-	-	-	0.31
38	14.480	1149	1143	Cadalane	$C_{12}H_{22}$	81	166, 151, 137, 123, 109, 95, 81, 67, 55, 41	-	-	-	-	1.76
39	14.608	1153	1149	1,3-Dimethyladamantane	$C_{12}H_{20}$	149	164, 149, 121, 107, 93, 79, 67, 55, 41	-	-	-	-	2.42

40	14.758	1158	1167	1,3-Dimethyladamantane	$C_{12}H_{20}$	149	164, 149, 121, 107, 93, 79, 67, 41	-	-	-	-	2.34
41	14.905	1162	1163	Cadinane	$C_{12}H_{22}$	81	166, 151, 123, 109, 95, 81, 67, 55, 41	-	-	-	-	1.01
42	15.174	1171	1173	Cadinane	$C_{12}H_{22}$	81	166, 151, 137, 123, 109, 95, 81, 67, 55, 41	-	-	-	-	2.13
43	15.381	1178	1173	Eudesmane	$C_{12}H_{22}$	81	166, 151, 137, 123, 109, 95, 81, 67, 55, 41	-	-	-	-	0.29
44	15.509	1182	1087	1,3-Dimethyladamantane	$C_{12}H_{20}$	149	164, 149, 121, 107, 93, 79, 67, 41	-	-	-	-	1.46
45	15.605	1185	1183	Cadinane	$C_{12}H_{22}$	81	166, 151, 137, 123, 109, 95, 81, 67, 55, 41	-	-	-	-	0.37
46	15.715	1188	1183	1-(2-Methyl-2-cyclopenten-1-yl)-cyclohexane	$C_{12}H_{18}$	106	162, 147, 133, 119, 106, 91, 67, 53	-	-	-	-	0.74
47	15.777-15.782	1190	1194	Dodecane	$C_{12}H_{26}$	57	112, 99, 85, 71, 57, 43, 41, 35	0.27	1.85	0.63	0.41	-
48	15.785	1191	1193	Cadalane	$C_{12}H_{22}$	95	166, 151, 137, 124, 109, 95, 81, 67, 55, 41	-	-	-	-	0.68
49	16.099	1201	1199	(-)-Carvone	$C_{10}H_{14}O$	82	150, 135, 117, 108, 93, 82, 58, 54, 39, 35	-	-	0.25	-	-
50	16.159	1203	1205	Tetramethyladamantane	$C_{14}H_{24}$	177	192, 177, 149, 135, 121, 107, 95, 79, 67, 55, 41	-	-	-	-	0.22

51	16.482	1214	1219	Selinane	$C_{12}H_{20}$	149	164, 149, 137, 121, 107, 93, 81, 67, 55, 41	-	-	-	-	0.23
52	16.656	1220	1215	1,4-Dimethyladamantane	$C_{12}H_{20}$	149	164, 149, 135, 121, 107, 93, 67, 55, 41	-	-	-	-	0.19
53	18.244	1273	1270	Farnesane	$C_{15}H_{32}$	57	212, 190, 179, 162, 155, 127, 99, 85, 71, 57, 43	-	-	-	-	0.12
54	18.709	1289	1283	Tritetracontane	$C_{43}H_{88}$	57	178, 155, 141, 127, 112, 99, 85, 71, 57, 43, 41, 35	-	-	0.32	0.22	-
55	21.475	1388	1390	Pentadecane	$C_{15}H_{32}$	57	212, 183, 169, 155, 141, 127, 113, 97, 85, 71, 57, 43, 41, 29, 15	-	-	0.41	0.34	-
56	21.740	1398	1392	Caryophyllene	$C_{15}H_{24}$	93	204, 189, 175, 164, 161, 147, 133, 119, 105, 93, 79, 69, 53, 41, 27, 15	-	-	0.15	0.16	-
57	23.159	1452	1450	2,6,10-Trimethyltridecane	$C_{16}H_{34}$	57	226, 211, 197, 183, 169, 155, 141, 127, 113, 99, 85, 71, 57, 43	-	-	-	0.25	-
58	23.408	1461	1469	Aromadendrene	$C_{15}H_{24}$	105	204, 189, 175, 161, 147, 133, 121, 105, 93, 79, 67, 55, 41	-	-	-	0.34	-
59	23.515	1465	1461	2-Hexyldecanol	$C_{16}H_{34}O$	111	197, 180, 165, 152, 137, 111, 85, 83, 71, 57, 43	-	-	-	0.15	-

60	23.849-23.866	1479	1472	2,6-Di-tert-butylphenol	$C_{14}H_{22}O$	57	206, 191, 175, 163, 147, 135, 107, 85, 71, 57, 41, 35	0.27	-	0.27	0.19	-
61	24.079-24.106	1487-1488	1489	Heneicosane	$C_{21}H_{44}$	133	169, 155, 141, 127, 113, 99, 85, 71, 57, 43, 41, 37	0.53	4.38	0.71	0.63	0.27
62	26.075	1567	1568	Carotol	$C_{15}H_{26}O$	161	222, 204, 189, 175, 161, 137, 119, 105, 97, 81, 69, 55, 41	-	-	-	-	0.23
63	26.351-26.355	1578	1579	Hexahydro-3-butylphthalide	$C_{12}H_{20}O_2$	139	196, 178, 168, 150, 139, 124, 112, 111, 93, 81, 67, 55, 43, 41, 29	-	-	0.17	0.20	-
64	26.533-26.545	1586	1588	Hexadecane	$C_{16}H_{34}$	108	169, 155, 141, 127, 113, 99, 85, 71, 57, 43, 41, 37	0.30	-	0.51	0.44	0.13
65	26.624-26.639	1589	1592	1-Ethyl-4,5-divinyl-cyclohexene	$C_{12}H_{18}$	107	150, 133, 121, 105, 94, 79, 65, 51, 41, 35	1.63	-	1.56	1.55	-
66	27.222	1613	1615	Butylidenephthalide	$C_{12}H_{12}O_2$	159	188, 159, 146, 131, 115, 103, 89, 77, 63	-	-	-	0.32	-
67	27.376-27.395	1621	1628	1,1-Dimethyl-1,2,3,5,7,8,9,9a-octahydro-benzocyclohepten-6-one	$C_{13}H_{20}O$	108	192, 163, 150, 135, 122, 108, 91, 79, 51, 41, 36	0.64	-	0.55	0.75	-
68	27.538	1627	1625	8-Hydroxy-2,2,8-trimethyldeca-5,9-dien-3-one	$C_{13}H_{22}O_2$	57	192, 167, 139, 108, 85, 79, 57, 55, 41, 35	-	-	-	0.17	-
69	28.165-28.170	1654	1657	Senkyunolide	$C_{12}H_{16}O_2$	55	192, 180,163, 145, 117, 91, 55	1.33	-	1.20	1.67	-

70	28.377-28.420	1663-1664	1667	Sedanolide	$C_{12}H_{18}O_2$	57	194, 165, 137, 123, 108, 9	12.13	9.07	11.70	10.95	0.41
71	28.602-28.609	1672-1673	1671-1675	Butylidenephthalide	$C_{12}H_{14}O_2$	57	190, 175, 161, 133, 105, 91	1.07	-	1.47	1.79	-
72	28.715	1677	1677	Sedanolide	$C_{12}H_{18}O_2$	108	194, 137, 124, 108, 91, 79, 53	-	-	-	0.29	-
73	28.868-28.889	1684-1685	1681	Heptadecane	$C_{17}H_{36}$	57	141, 127, 113, 99, 85, 71, 57, 43, 41, 36	0.33	-	0.95	0.49	-
74	29.251-29.273	1700-1701	1705	Eicosane	$C_{20}H_{42}$	68	141, 127, 113, 99, 85, 71, 57, 43, 41, 36	0.34	4.08	0.24	0.26	0.17
75	31.091-31.111	1783	1780	Octadecane	$C_{18}H_{38}$	70	141, 127, 113, 99, 85, 71, 57, 43, 41, 37	0.23	-	0.49	0.32	0.14
76	31.357	1794	1794	Isopropyl myristate	$C_{17}H_{34}O_2$	43	270, 228, 211, 199, 185, 171, 157, 143, 129, 115, 102, 85, 73, 43	-	-	-	-	0.28
77	31.715-31.730	1812	1814	6,10,14-Trimethyl-2-pentadecanone	$C_{18}H_{36}O$	43	268, 250, 235, 225, 210, 194, 179, 165, 152, 140, 124, 109, 95, 85, 71, 58, 43, 29	-	-	0.28	0.26	-
78	31.877-31.920	1819-1821	1819	Neophytadiene	$C_{20}H_{38}$	82	137, 123, 109, 95, 82, 68, 41, 36	15.21	18.91	22.28	26.43	0.49
79	32.080-32.095	1828-1829	1820	Phytene	$C_{20}H_{40}$	82	140, 125, 111, 85, 70, 55, 41, 37	0.30	-	0.39	0.38	-
80	32.364-32.383	1842-1843	1843	Neophytadiene	$C_{20}H_{38}$	149	151,137, 123, 109, 95, 82, 57, 43, 41, 35	1.05	-	1.18	1.14	-

81	32.745-32.766	1860-1861	1862	Neophytadiene	$C_{20}H_{38}$	57	152,137, 123, 109, 95, 82, 57, 43, 41, 36	1.66	2.48	2.00	1.83	-
82	33.225	1882	1887	Nonadecane	$C_{19}H_{40}$	57	268, 239, 225, 211, 197, 183, 169, 155, 141, 127, 113, 99, 85, 71, 57, 43, 41, 35	-	-	0.51	-	-
83	33.395	1890	1895	Methyl palmitate	$C_{17}H_{34}O_2$	74	270, 239, 227, 213, 199, 185, 171, 157, 143, 129, 115, 101, 87, 74, 57, 41	-	-	-	-	0.18
84	33.535-33.538	1897	1898	1,2-Benzenedicarboxylic acid, butyl 8-methylnonyl ester	$C_{22}H_{34}O_4$	67	150,149,135, 121, 104, 93, 70, 55, 41, 35	0.26	-	0.27	0.24	-
85	33.893	1914	1916	Eicosane	$C_{20}H_{42}$	55	197, 183, 169, 155,141, 127, 113, 99, 85, 71, 57, 43, 41, 35	0.36	-	-	-	-
86	34.145	1927	1928	Palmitic acid	$C_{16}H_{32}O_2$	73	256, 239, 227, 213, 199, 185, 171, 157, 129, 115, 97, 73, 57, 43	-	-	-	-	0.43
87	35.225	1981	1983	Heneicosane	$C_{21}H_{44}$	57	296, 281, 267, 253, 239, 225, 211, 197, 183, 169, 155, 141, 127, 113, 99, 85, 71, 57, 43, 41, 27	-	-	0.37	0.28	-
88	36.598	2050	2053	Methyl linoleate	$C_{19}H_{34}O_2$	67	294, 262, 178, 164, 150, 123,	-	-	-	-	0.26

							109, 95, 81, 67, 55, 41					
89	36.609	2051	2056	Z,E-2,13-Octadecadien-1-ol	$C_{18}H_{34}O$	71	178, 164, 150, 127, 109, 95, 81, 67, 55, 41, 35	0.63	-	-	-	-
90	36.780	2060	2060	Methyl vaccenate	$C_{19}H_{36}O_2$	84	264, 222, 197, 180, 166, 137, 123, 97, 74, 69, 55, 41, 35	0.25	-	-	-	0.29
91	36.940	2068	2065	2-(5-Oxohexyl)-cyclopentanone	$C_{11}H_{18}O_2$	84	179, 163, 149, 139, 121, 111, 84, 83, 57, 43, 41, 36	-	-	-	0.26	-
92	37.135	2078	2075	Phytol	$C_{20}H_{40}O$	57	296, 278, 123, 95, 71, 57	3.54	4.65	4.06	7.80	-
93	37.564	2101	2105	2-(5-Oxohexyl)-cyclopentanone	$C_{11}H_{18}O_2$	43	178, 161, 139, 121, 111, 84, 83, 57, 41, 35	0.31	-	0.41	0.26	-
94	39.018	2179	2179	Heneicosane	$C_{21}H_{44}$	57	296, 281, 267, 253, 239, 225, 211, 197, 183, 169, 161, 148, 136, 113, 99, 85, 71, 57, 43, 41, 35	0.24	-	0.28	-	0.11
95	39.331	2195	2195	Glycidyl palmitate	$C_{19}H_{38}O_4$	43	256, 239, 196, 182, 168, 98, 84, 57, 43, 41	-	-	-	0.20	0.14
96	40.768	2277	2275	Heneicosane	$C_{21}H_{44}$	57	239, 197, 183, 169, 155, 141, 127, 113, 99, 85, 71, 57, 43, 41, 35	0.28	-	0.26	0.17	0.20
97	42.174-42.197	2358	2359	Glyceryl linoleate	$C_{21}H_{38}O_4$	57	262, 234, 191, 178, 164, 149,	0.38	-	0.25	0.34	0.19

							135, 121, 110, 95, 81, 67, 55, 41, 35					
98	42.483-44.116	2376-2474	2379	Tetracosane	$C_{24}H_{50}$	57	141, 127, 113, 99, 85, 71, 57, 43, 41, 35	0.37	3.15	0.36	-	0.42
99	42.621-42.645	2384-2385	2388	Eicosanal	$C_{20}H_{40}O$	59	152, 138, 124, 110, 96, 82, 57, 43, 41, 35	0.63	-	0.55	0.41	-
100	43.125	2413	2415	Glycidyl oleate	$C_{20}H_{36}O_3$	55	324, 295, 185, 151, 129, 116, 81, 69, 55, 41	-	-	-	-	0.13
101	43.687	2449	2451	*n*-1-Tetracosanol	$C_{24}H_{50}O$	57	181, 167, 153, 139, 125, 111, 97, 83, 55, 41, 37	7.20	5.80	4.43	3.72	-
102	43.984	2467	2466	2-Nonadecanone	$C_{19}H_{38}O$	149	152, 125, 111, 85, 71, 59, 43, 41, 35	0.85	-	0.79	0.54	-
103	44.124	2474	2475	Pentacosane	$C_{25}H_{52}$	57	155, 141, 127, 113, 99, 85, 71, 57, 43, 41, 35	0.55	-	-	-	0.45
104	45.301	2550	2555	Octacosanol	$C_{28}H_{58}O$	57	169, 153, 139, 125, 111, 97, 83, 57, 55, 41, 37	0.31	-	-	0.17	-
105	45.685	2573	2570	Tetracosane	$C_{24}H_{50}$	57	211, 197, 183, 169, 155, 141, 127, 113, 99, 85, 71, 57, 43, 41, 35	0.50	3.47	0.47	0.14	0.55
106	45.905	2586	2585	Eicosanal	$C_{20}H_{40}O$	82	152, 138, 124, 110, 96, 82, 57, 43, 41, 35	1.22	2.34	0.98	0.79	1.85

107	46.879-46.892	2650-2651	2650	1-Heptacosanol	$C_{27}H_{56}O$	57	167, 153, 139, 125, 111, 97, 83, 55, 41, 35	8.24	5.95	5.03	4.51	0.27
108	47.149	2668	2666	2-Nonadecanone	$C_{19}H_{38}O$	59	152, 131, 111, 85, 71, 59, 43, 41, 35	1.36	-	1.37	0.89	0.47
109	48.395	2752	2750	Octacosanol	$C_{28}H_{58}O$	57	167, 153, 127, 125, 111, 97, 83, 57, 43, 41, 36	0.39	-	-	0.22	-
110	48.661	2770	2774	Tetracontane	$C_{40}H_{82}$	57	337, 323, 309, 295, 281, 267, 253, 239, 225, 211, 197, 183, 169, 155, 141, 127, 113, 99, 85, 71, 57, 43, 41	-	-	-	-	0.37
111	48.796	2781	2780	Squalene	$C_{30}H_{50}$	69	410, 137, 95, 81, 69	0.28	-	-	0.33	0.34
112	48.908	2788	2789	Hexacosanal	$C_{26}H_{52}O$	82	152, 138, 124, 110, 96, 82, 57, 43, 41, 35	1.90	-	1.48	1.14	0.39
113	49.823-49.849	2852-2853	2853	Octacosanol	$C_{28}H_{58}O$	57	153, 139, 125, 111, 97, 83, 57, 55, 41, 36	9.68	4.54	6.57	5.67	1.11
114	47.203-50.085	2672-2870	2674	Tetracontane	$C_{40}H_{82}$	57	183, 169, 155, 141, 127, 113, 99, 85, 71, 57, 43, 41, 38	0.60	2.74	0.51	0.38	0.38
115	50.268	2884	2883	Methyl hexacosanoate	$C_{27}H_{54}O_2$	74	281, 255, 221, 199, 185, 171, 157, 143, 127, 115, 101, 87, 74, 57, 43, 41, 37	0.65	-	-	0.44	-

116	51.735	2990	2993	Octacosanal	$C_{28}H_{56}O$	82	416, 390, 255, 228, 203, 180, 159, 138, 109, 96, 82, 57, 43, 41, 37	0.61	-	0.55	0.46	-
117	52.585	3052	3047	Octacosanol	$C_{28}H_{58}O$	57	167, 153, 139, 125, 111, 97, 83, 57, 43, 41, 36	1.02	-	0.78	0.67	-
118	52.758	3065	3065	Tetracontane	$C_{40}H_{82}$	57	489, 417, 379, 355, 323, 295, 267, 239, 127, 99, 85, 71, 57, 43, 41	-	-	-	-	0.14
119	52.790	3067	3069	α-Tocopherol (vitamin E)	$C_{29}H_{50}O_2$	59	205, 165, 152, 121, 109, 85, 71, 59, 43, 41, 37	-	-	1.16	1.06	-
120	54.089	3162	3162	Campesterol	$C_{28}H_{48}O$	57	400, 382, 367, 354, 315, 289, 273, 213, 187, 145, 57, 43	-	-	-	0.24	-
121	54.576-54.891	3197-3200	3197	Stigmasterol	$C_{29}H_{48}O$	55	412, 394, 379, 369, 351, 314, 300, 285, 271, 255, 241, 229, 213, 199, 145, 133, 123, 105, 95, 83, 69, 55, 41, 38	-	3.87	4.34	3.39	-
122	55.476-55.515	3263-3266	3263	γ-Sitosterol	$C_{29}H_{50}O$	43	414, 396, 381, 354, 329, 303, 273, 255, 231, 213, 199, 185, 173, 161, 145,	-	-	4.34	0.43	-

							133, 119, 107, 95, 81, 57, 43, 41, 36					
123	56.565-56.600	3343-3346	3348	Stigmasterone	$C_{29}H_{46}O$	55	410, 367, 349, 312, 298, 283, 271, 245, 227, 215, 203, 187, 175, 161, 147, 133, 123, 109, 95, 81, 69, 55, 41	-	-	0.49	0.39	-

Putative identifications were based on NIST mass spectral library matching, characteristic fragmentation patterns, and agreement between experimental and literature retention indices. No authentic reference standards were analyzed.

CS: Soil-grown celery; CA: Aquaponically grown celery without Spirulina supplementation; CAS2, CAS4, and CAS8: Aquaponically grown celery supplemented with Spirulina at 2, 4, and 8 g L^{-1}, respectively

.

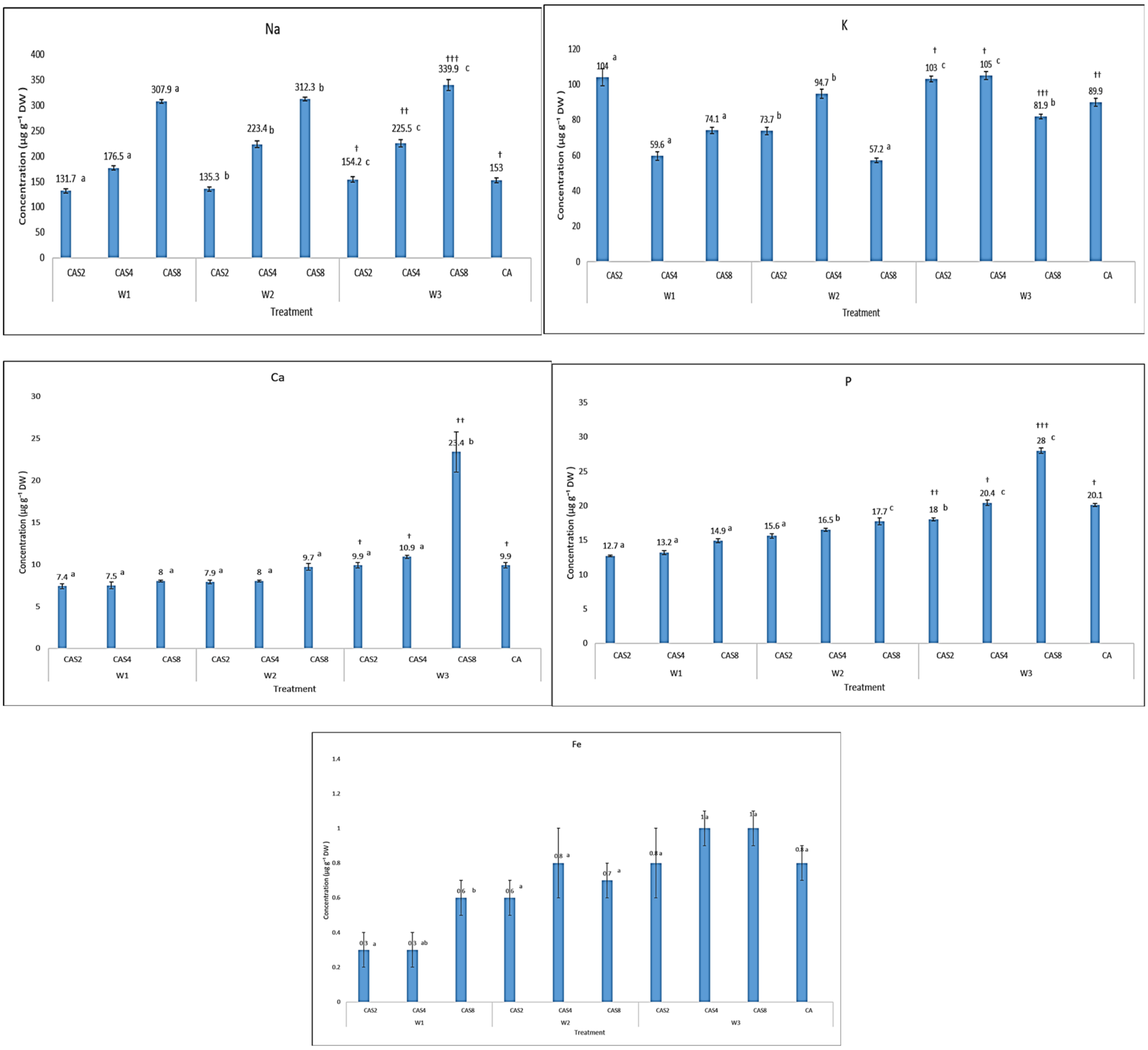

Figure S1. Mineral content of aquaponically cultivated celery following Spirulina supplementation. Na, K, Ca, Fe, and P were measured after one (W1), two (W2), and three (W3) weeks of supplementation with 2, 4, and 8 g L^{-1} Spirulina. Values are presented as mean ± SD (n = 3 independent tank-level biological replicates). Different letters within each week indicate significant differences among Spirulina doses based on Tukey-adjusted simple-effect comparisons following a significant Spirulina dose × week interaction ($p < 0.05$). The longitudinal analysis included CAS2, CAS4, and CAS8 only because CA was sampled only at week 3

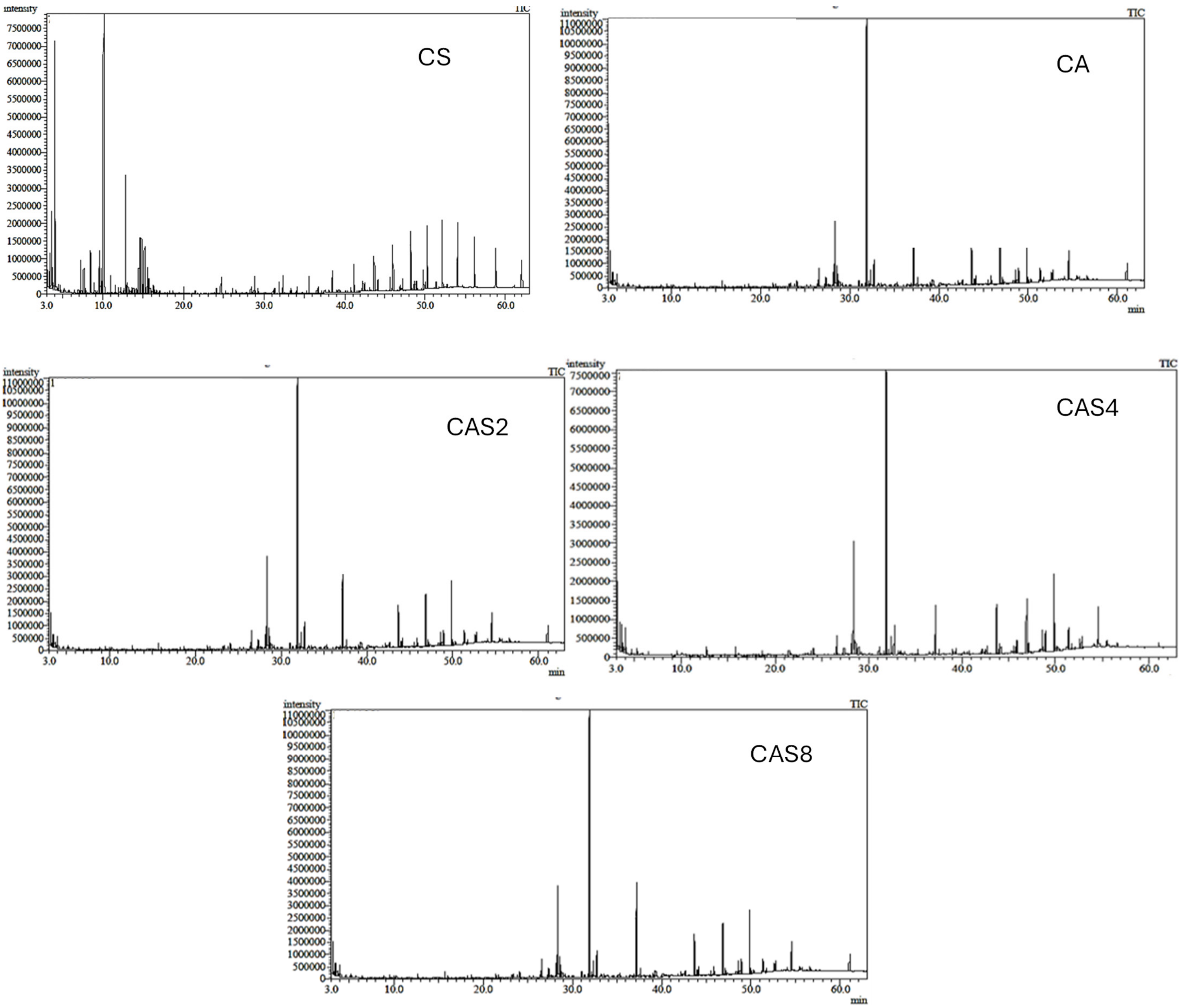

Figure S2. Representative GC–MS chromatograms of metabolites detected in soil-grown celery (CS), untreated aquaponic celery (CA), and Spirulina-treated aquaponic celery (CAS2, CAS4, and CAS8). The chromatograms are presented for descriptive chemical profiling only; because the GC–MS analysis was not independently biologically replicated, no inferential statistical analysis was performed. Compound assignments were considered putative based on mass-spectral library matching and retention-index agreement and were not confirmed using authentic reference standards.

Chemical class	Compound name	CS	CA	CAS2	CAS4	CAS8
Hydrocarbons	trans-1,3-Dimethylcyclohexane	3.41	0.92	0	2.25	1.13
	1,3-Dimethyladamantane	11.52	0	0	0	0
	Octane	8.19	0.58	0	1.31	0.62
**Total major hydrocarbons**		23.12	1.5	0	3.56	1.75
Sesquiterpenes	Cadinane	3.51	0	0	0	0
**Total major sesuiterpenes**		3.51	0	0	0	0
Monoterpenes & oxygenated monoterpenes	Camphene	1.23	0	0	0	0
	α-Pinene	2.8	0	0	0	0
	p-Cymene	1.09	0	0	0	0
	D-Limonene	12.66	0	0	0	0
	Eucalyptol (1,8-cineole)	3.82	0	0	0	0
**Total major monoterpenes and oxygenated monoterpenes**		21.6	0	0	0	0
Phthalides	Senkyunolide	0	1.33	0	1.2	1.67
	Sedanolide	0.41	12.13	9.07	11.7	11.24
	Butylidenephthalide	0	1.07	0	1.47	2.11
**Total major phthalides**		0.41	14.53	9.07	14.37	15.02
Diterpenes / Diterpene alcohols	Neophytadiene	0.49	17.92	21.39	25.46	29.4
	Phytol	0	3.54	4.65	4.06	7.8
**Total major diterpenes and diterpene alcohols**		0.49	21.46	26.04	29.52	37.2
Long-chain fatty alcohols	n-Tetracosanol	0	7.2	5.8	4.43	3.72
	1-Heptacosanol	0.27	8.24	5.95	5.03	4.51
	Octacosanol	1.11	11.4	4.54	7.35	6.73
**Total major long-chain fatty alcohols**		1.38	26.84	16.29	16.81	14.96
Phytosterols & antioxidant	α-Tocopherol	0	0	0	1.16	1.06
	Stigmasterol	0	0	3.87	4.34	3.39
	γ-Sitosterol	0	0	0	4.34	0.43
**Total major phytosterols and antioxidant**		0	0	3.87	9.84	4.88

Relative peak areas of the same compound detected in multiple peaks were summed to facilitate comparison
Color intensity represents normalized relative metabolite abundance based on GC–MS peak area percentages.

Figure S3. Heatmap showing relative abundance of individual metabolites identified by GC–MS in soil-grown celery (CS), untreated aquaponic celery (CA), and Spirulina-treated aquaponic celery (CAS2, CAS4, and CAS8). The heatmap represents descriptive relative peak-area patterns; no inferential statistical analysis was performed on the GC–MS dataset. GC–MS results are descriptive only.

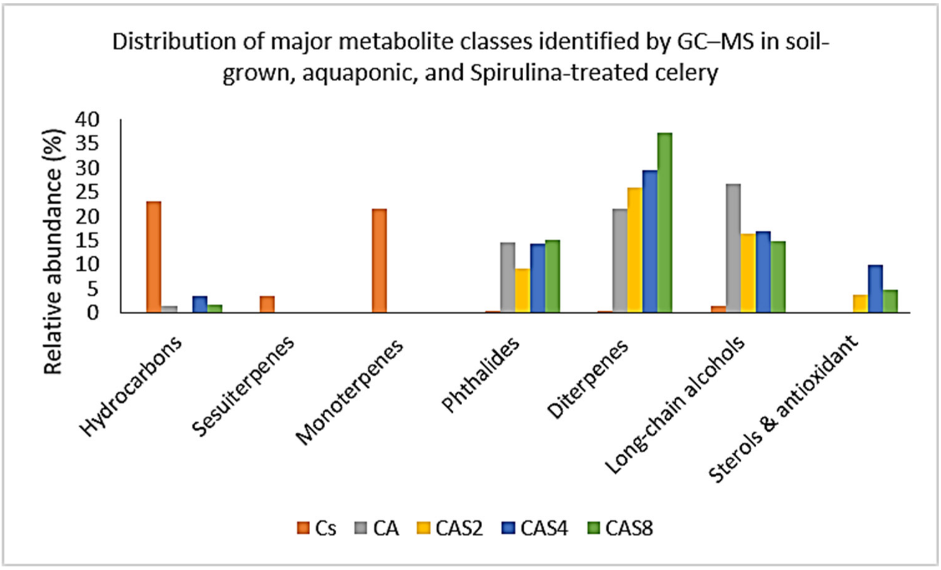

Figure S4. Distribution of major metabolite classes identified by GC–MS in soil-grown, untreated aquaponic, and Spirulina-treated aquaponic celery. Values represent descriptive relative GC–MS peak-area percentages from the analyzed samples. No inferential statistical analysis was performed on the GC–MS dataset.

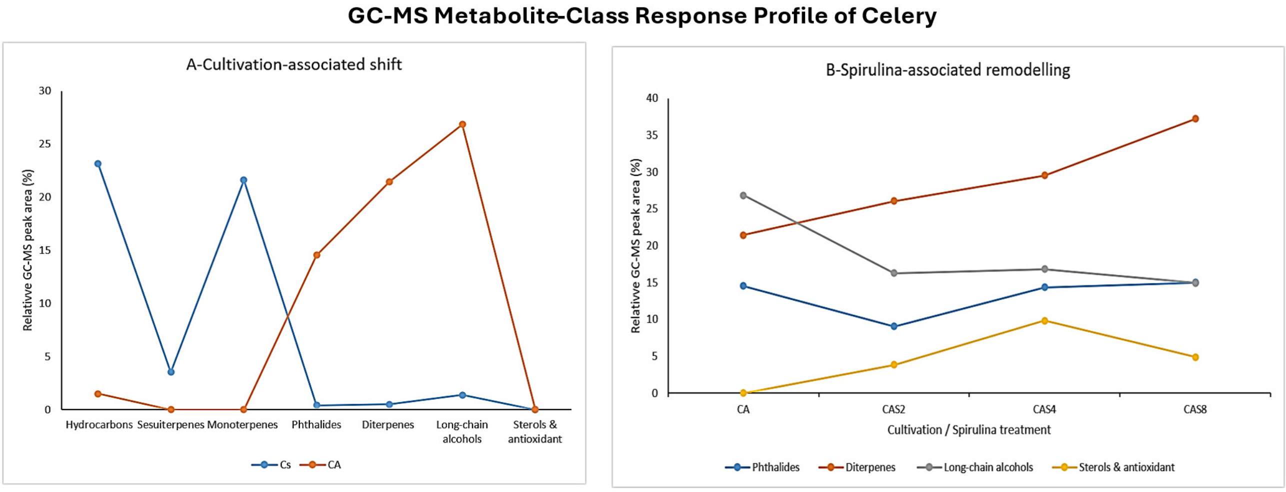

Figure S5. GC–MS metabolite-class response profile of soil-grown and aquaponically cultivated celery and the observed differences associated with Spirulina supplementation. The profile is descriptive and does not represent statistical treatment effects. No inferential statistical analysis was performed on the GC–MS dataset.
